# Identification of a m6A-immune-related risk model for predicting prognosis, immune microenvironment, and drug responses in acute myeloid leukemia

**DOI:** 10.1038/s41598-025-22002-5

**Published:** 2025-11-03

**Authors:** Yanliang Bai, Huijie Nan, Lijie Wang, Peiyao Yang, Yabin Cui, Jinhui Xu, Mingyue Shi, Yuqing Chen

**Affiliations:** 1 Department of Hematology, Zhengzhou University People’s Hospital and Henan Provincial People’s Hospital, Zhengzhou, Henan China; 2 Department of Hematology, Henan University People’s Hospital and Henan Provincial People’s Hospital, Zhengzhou, Henan China

**Keywords:** m6A (N6-methyladenosine), Immune, TME, Prognosis, Acute myeloid leukemia, Treatment, Acute myeloid leukaemia, Leukaemia

## Abstract

This study utilized TCGA database to explore the role of m6A modification and immune infiltration in AML. Through unsupervised clustering and WGCNA analysis, 8 hub genes were identified, and a risk model with *EHBP1L1* and *ZNF385A* was established using LASSO regression. A nomogram incorporating hub gene risk score and age showed satisfactory prognostic prediction. External validation of GEO confirmed the model’s effectiveness. TME analysis revealed correlations with monocytes and Treg cells, while immune checkpoints and HLA genes were associated with risk scores. Drug sensitivity analysis suggested potential responses to specific chemotherapy drugs. TIDE analysis indicated reduced ICI treatment benefit in high-risk patients. RT-qPCR validations revealed the significance of prognosis and risk stratification of *ZNF385A*. The noticeable trend of *EHBP1L1* was observed. In addition, the accurate predictive capability of the risk model has been validated by clinical samples. Therefore, the risk model enables a quantitative evaluation of disease severity and progression risk in AML patients, based on their clinical and biological characteristics. This precise prediction not only informs treatment decisions but also guides the selection of chemotherapy regimens, overall improving patient outcomes.

**Supplementary Information:**

The online version contains supplementary material available at 10.1038/s41598-025-22002-5.

## Introduction

Acute myeloid leukemia (AML) is recognized as one of the most aggressive hematological malignancies, with the highest incidence in adults^1^. Despite advancements in treatment, the prognosis remains dismal, as most patients experience disease recurrence or develop resistance to chemotherapy, even with targeted therapy or hematopoietic stem cell transplantation (HSCT)^2^. The long-term overall survival (OS) rate of young patients (< 60 years) is less than 40%, and that of elderly patients (≥ 60 years) is only 15%^3^. The development of AML is not only related to gene mutation and chromosomal variation, but also abnormal epigenetic regulation, such as DNA methylation and histone modification^4^.

RNA methylation, particularly N6-methyladenosine (m6A), stands at the forefront of current epigenetic research. This modification, the most prevalent internal mRNA mark, has been shown to influence mRNA stability, alternative splicing, nuclear export, and translation efficiency^5,6^. Recent studies have demonstrated that m6A regulators contributed to tumorigenesis, progression, and drug-resistance in AML by regulating specific coding and noncoding gene targets and signaling pathways^7,8^. For instance, m6A “eraser” *FTO* is highly expressed in refractory t (8;21) AML. *FTO* knockdown mediates *IGFBP2* overexpression and regains Ara-C tolerance in t (8;21) AML cells^9^. In addition, BMMSCs-derived (bone marrow mesenchymal stem cells, BMMSCs) *FTO*-*exo* enhanced AML progression and chemo-resistance by modulating the LncRNA GLCC1-IGF2BP1-c-Myc signal pathway^10^. *ALKBH5*, another m6A eraser, is required to keep AML stem cell activity intact^11,12^. In AML BMMSCs, *METTL3* expression is notably decreased. This reduction in expression mediates adipogenesis in BMMSCs, consequently fostering chemoresistance in human AML cell lines in vitro.^13^. *YTHDF2* is aberrantly overexpressed in patients with AML, particularly at relapse. *YTHDF2* promotes the expression of miR-126-3p to promote acute myeloid leukemia progression. Researchers identified the role of *YTHDC1* on self-renewal of leukemic stem cells in AML^14,15^. Collectively, these findings indicated that m6A regulators play an important role in maintaining leukemia stem cells and induce drug resistance in AML. Therefore, m6A not only plays an important role in maintaining leukemia stem cells and inducing drug tolerance in AML, but also has good prognosis and therapeutic potential.

Recent evidence increasingly suggests that m6A regulators are closely linked to immune evasion in cancer and exert a crucial influence on the tumor microenvironment (TME), immune recognition, and immune response^16,17^. The TME is acknowledged as a complex ecosystem consisting of cancer cells, immune cells, stromal cells, and various non-cellular components. Each component is considered to play a critical role in regulating tumor progression^18^. *METTL3*, a core component of the m6A writer complex, has been reported to affect both cancer cells and the surrounding TME. Li et al. found that depletion of *METTL3* impaired mouse T cell homeostasis and differentiation^19^. Suppression of the *METTL3*-m6A-integrin β1 axis impairs T cell infiltration and antitumor activity^20^. The loss of *YTHDF2* in regulatory T (Treg) cells reduces tumor growth in mice. *YTHDF2* modified transcripts that encode NF-κB, and controlled anti-tumor immunity by regulating intra-tumoral Tregs^21^. Han et al. reported higher expression levels of NK cells and CD8 + cytotoxic T cells in the tumors of *YTHDF1* knockout mice compared to WT mice. This finding suggests a stronger antitumor response in the presence of *YTHDF1*^22–24^. However, the mechanism of m6A methylation in TME is still little understood. Most of the studies mainly focus on the impact of m6A regulators on TME and construct a risk model based on m6A modification^25–29^. Rare studies have explored the mutual effect of immune-related genes on m6A methylation. Therefore, a comprehensive investigation of m6A regulators in immune-related genes, as well as the prognostic value and the risk score in immunotherapy and chemotherapy, will provide a more comprehensive understanding of the interaction of m6A and TME.

This study explored the genes related to m6A and immunity in AML, and performed clinical risk modeling to evaluate the survival prognosis of AML patients. Our findings provide a detailed understanding of m6A-immune compositions in AML and TME changes in AML with poor prognosis. Herein, it is demonstrated that m6A regulators play an indispensable role in TME and contribute to making therapeutic strategies for AML. Finally, we verified the expression of hub genes and immune checkpoint genes in clinical AML samples by using quantitative reverse transcription polymerase chain reaction (RT-qPCR).

## Materials and methods

### Isolation of bone marrow cDNA

The samples of 20 de novo AML patients were collected at Zhengzhou University People’s Hospital. For these patients, a sufficient amount of RNA from tumor biopsies was extracted for RT-qPCR. Details on AML patient samples are shown in Table S1. The study was approved by the Committee for the Ethical Review of Research, Zhengzhou University People’s Hospital and informed consent was obtained from all the patients. The patients/participants [legal guardian/next of kin] provided written informed consent to participate in this study. All methods were performed in accordance with the relevant guidelines and regulations.

### AML dataset acquisition and processing

**Fig. 1:**
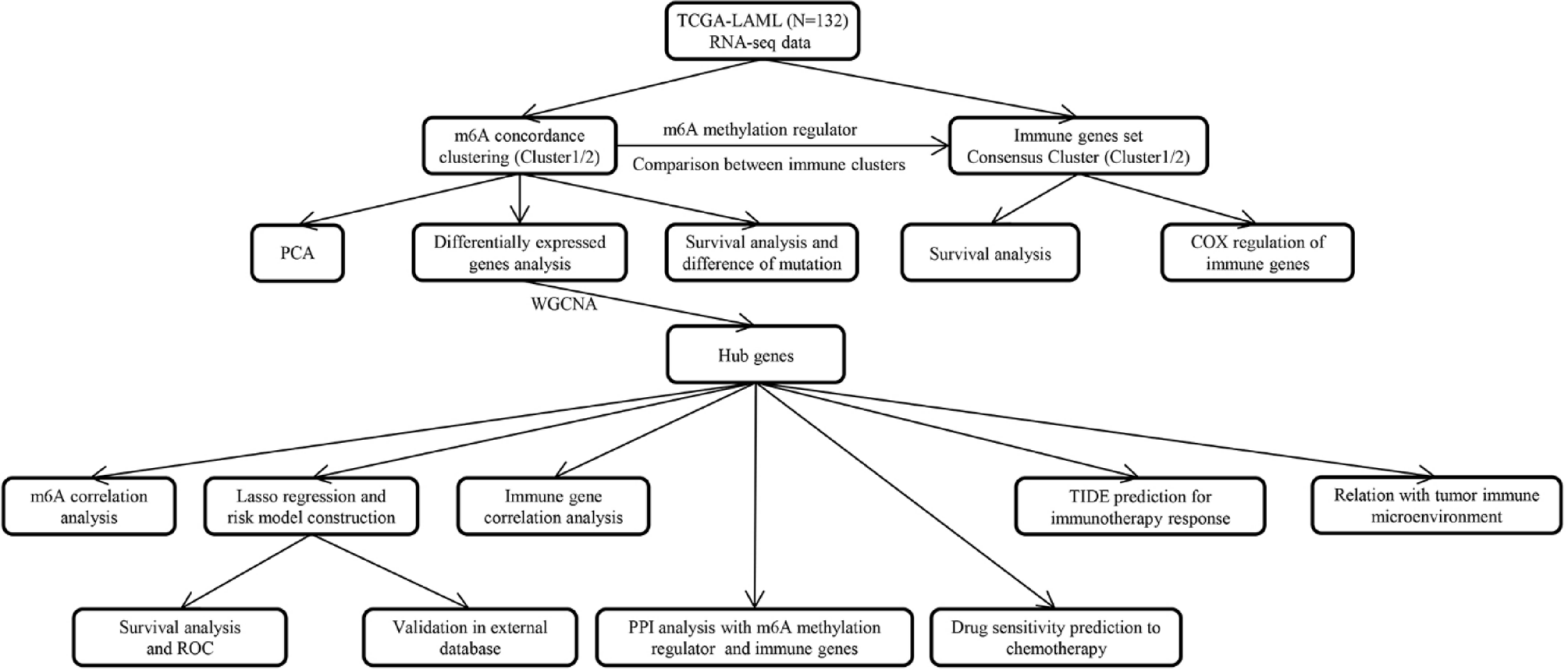
Schematic diagram of the major process in the present study.

We acquired the RNA sequencing (RNA-seq) data and relevant clinical profiles of AML patients from The Cancer Genome Altas (TCGA) database. Before the analysis, genes that expressed 0 in more than 30% of the samples and samples without survival information were removed, finally, 132 samples remained. The relevant clinicopathological information was downloaded for further analysis. The clinical characteristics for AML cases were summarized in Table S2. Data from the eligible Gene Expression Omnibus (GEO, GSE23143) dataset were used for deeper assays. The Xena browser at the University of California, Santa Cruz (UCSC) provided the somatic mutation datasets (n = 151) for download (https://xenabrowser.net/datapages/). Figure 1 depicts the current study’s methodology. The Declaration of Helsinki was followed in the conduct of the study.

### Unsupervised consensus molecular clustering for 21 m6A regulators

Previous studies have identified 21 m6A-related regulators^30^. We retrieved 21 m6A gene expression matrices from the TCGA AML cohort. The 21 m6A RNA methylation regulators from published literature (including erasers *ALKBH5* and *FTO*, writers *ZC3H13, ZC3H13, KIA1429, RBM15, RBM15B, METTL3, METTL14, METTL16* and *WTAP*), and readers (*RBMX, HNRNPA2B1, HNRNPC, IGF2BP1, IGF2BP2, IGF2BP3, YTHDC1, YTHDC2, YTHDF1, YTHDF2* and *YTHDF3*). Based on the expression of the 21 m6A RNA methylation regulators in AML, an unsupervised cluster assay was employed to determine different m6A modification patterns and to categorize patients for more in-depth assays. Consensus Cluster Plus software and R Bioconductor software were employed to implement the gene expression patterns and the survival differences in different subgroups based on the expression profiles of m6A methylation regulators.

### Differentially expressed genes (DEGs) and function analysis of m6A cluster subgroups

Based on the expression of the 21 m6A modulators, the patients were categorized into 3 distinct m6A modification pattern groups in order to determine the m6A-associated genes. To distinguish between various mutation patterns, DEGs were found using the seasoned “limma” R package. Additionally, DEGs were retrieved from genes having an adj. *P* Value < 0.01 and a log FC ≥ 1. We downloaded the Hallmark gene set in the M Sig DB database (https://www.gsea-msigdb.org/gsea/msigdb), and used Gene Set Variation Analysis (GSVA) R software to perform the gene enrichment analysis of the DEGs in different m6A modification patterns. Then, using the “limma” software to identify the differentially expressed pathways enriched in different m6A modification patterns.

### Tumor mutation burden (TMB) calculation

TMB refers to the number of non-synonymous mutations per Mb. The more mutations means that the more antigens might be generated, which makes tumor cells more likely to be recognized by the immune system and more sensitive to immunotherapy. We calculated the number of non-synonymous mutations (including missense mutations and nonsense mutations) in each sample. The size of the all-exome chip in TCGA is about 38 MB. We used the non-synonymous mutations number/38 Mb to get the TMB of each sample.

### Unsupervised clustering of immune-related genes

We downloaded the immune-related gene set in the Innate DB (https://www.innatedb.ca/) database and then matched the gene set with the pre-processed RNA expression profile of LAML to obtain the immune-related gene expression profile. The expression profiles of the immune gene sets were clustered unsupervised (k-mean clustering, k = 2), and then the R package “survival” was used to characterize the survival differences between immune subgroups. Next, one-way Cox regression analysis of immune genes was performed to assess the effect of immune gene expression on prognosis. Finally, the R package “ggpubr” was used to analyze the differences between the 21 m6A methylation regulators in the immune gene clustering groups (the Wilcoxon rank sum test was chosen).

### The WGCNA of DEGs among m6A subgroups

The R package “WGCNA” was used to analyze the weighted correlation network of the DEGs (4148) among m6A subgroups. First, the DEGs were clustered and outlier samples were excluded. Then the optimal soft threshold value (R2 reached 0.85) was selected. To divide the genes into individual modules, the minimum module size was set to 30 and the cut height was set to 0.2. The modules most relevant to the m6A methylation cluster and the immune gene clustering cluster were selected as the key modules. Hub genes were defined as those with gene significance (GS) > 0.6 and module membership (MM) > 0.7 in the key modules.

### Correlation analysis of hub genes with m6A methylation regulators and immune-related genes

The correlations and *P* values of the hub gene with 21 m6A methylation regulators were analyzed by “Hmisc”. The correlations and *P* values of hub genes with 884 immune-related genes were analyzed by “Hmisc”. *P* values < 0.05 were considered to be correlated.

### Lasso regression analysis screens for key prognosis-related genes

The effect of high and low expression levels of the hub gene on prognosis was analyzed using the R package “survival”. Lasso Cox regression analysis of the hub gene was performed using the R package “glmnet” to select clinical risk profiles of prognosis-related genes based on the best lambda values. The coefficients obtained from the Lasso regression were utilized to compute the risk score for each patient. In this calculation, N represents the number of prognostic genes, exp_i_ represents the expression level of the i-th gene, and β_i_ represents the regression coefficient of the i-th gene in the Lasso algorithm.

$${\text{Risk score = }}\mathop \sum \limits_{i = 1}^{n} {\text{exp}}_{i} *\beta_{i}$$

The sample was divided into a high-risk score group and a low-risk score group using the median of the risk scores. Using the R package “survival” Kaplan–Meier assessed the difference in survival time between the two groups. Finally, the R package “survival ROC” was used to compare the prognostic performance of the risk score in predicting 1-, 3-, and 5-year survival.

### Risk score combined with clinical parameters to build prognostic prediction models

Risk score and clinical parameters were used as independent variables and combined with time-to-death outcomes to perform univariate COX regression analysis. The best cut-off for age was analyzed using “Xtile” software, and age was converted to a categorical variable for univariate analysis. The cph function of the R package “rms” was used to incorporate the single-factor variables into the multi-factor COX regression model, and then the step function was used to filter the variables that were meaningful in the multi-factor model to construct the final prognostic prediction model. The final prognostic model was visualized as a column line graph using the nomogram function. The R package “rms” was used to calculate the C-index of the line plot model, and the probability of death score was calculated for each patient based on the probability of death obtained from the line plot. Based on the median values of the probability of death scores, all patients were divided into nomogram-high-risk and nomogram-low-risk groups. The time-survival profiles of the two groups were plotted as KM curves, and the survival differences between the two groups were analyzed. The R packages “ROC” and “time ROC” were used to compare the prognostic performance of the prognostic model in predicting 1-, 3-, and 5-year survival. The calibration curves at 1/3/5 years were plotted using the calibrate function of the R language “rms” package.

### External validation of the risk score and prognostic prediction model

The expression profile, clinical information, and survival information of AML sequencing data GSE23143 were downloaded from the GEO website. Using the prediction function of the R package “rms”, we calculated the death risk score for each patient by combining the hub gene expression and age, divided the patients into high- and low-risk groups, and plotted the survival curves. Based on the calculated probability death scores for each patient, combined with the survival outcomes of all patients at 1/3/5 years, ROC curves for predicting death at 1/3/5 years were plotted using the R packages “pROC” and “timeROC”. The calibration curves at 1/3/5 years were plotted using the calibrate function of the R language “rms” package.

### Tumor microenvironment (TME) cell infiltration analysis

CIBERSORTx website (http://cibersortx.stanford.edu/) was used to calculate the abundance of 22 immune cell types in the GSE25902^31^. Heatmaps of immune cell abundance were performed by the “pheatmap” R package. Using the “inferential statistics” by bulk RNA-seq. Based on a gene expression signature set of 22 immune cell subtypes provided by CIBETSORT website. The immune cell composition of patients in the high-risk and low-risk groups of the test set was measured based on this feature set.

### Immune checkpoint genes and HLA family genes analysis

Referring to the list of immune checkpoint genes and HLA family genes associated with immune characteristics used in the literature PMC8294515^32^, the expression of these genes was analyzed and compared between patients in the high-risk and low-risk groups. The correlation of Hub Gene risk scores with these gene expressions was analyzed.

### Immunotherapy response prediction analysis

TIDE (http://tide.dfci.harvard.edu/) represents tumor immune dysfunction and rejection. TIDE analysis results for the TCGA-AML cohort were obtained at the TIDE website for correlation analysis with the Hub Gene risk score in this study, as well as for comparison of TIDE analysis results (TIDE score, dysfunction score, and exclusion score) in high- and low-risk groups.

### Sensitivity analysis of chemotherapeutic drugs

Using the R package “oncoPredict”, the IC50 of several hundred chemotherapeutic drugs was predicted from the gene expression profile of each patient. The sensitivity of each patient to these chemotherapeutic drugs was then assessed and the five drugs that showed the strongest positive and negative correlation were selected. Commonly used chemotherapeutic agents for AML and popular drugs were also selected for analysis.

### Quantitative reverse transcription polymerase chain reaction (RT-qPCR)

Total RNA was isolated from the 20 de novo AML samples. The RNAeasy ™ Animal RNA Extraction Kit (Centrifuge Column; Beyotime; R0027) was used to extract total RNA from 20 AML samples (including favorable, intermediate and adverse). RNA detected by spectrophotometer (Thermo Scientific; NanoDrop 2000C). After genome removal, first-strand cDNA synthesis was achieved with reverse transcription using SweScript RT II First Strand cDNA Synthesis Kit (With gDNA Remover; Servicebio; G3333). Then, quantitative PCR was performed by SYBR® Select Master Mix (2X) (ABI; 4472908). The relative expression of m6A regulators was normalized to GAPDH and calculated by 2−ΔCt method. Primers sequences are listed in Table S3.

### Statistical analysis

R version 4.1.0 software was mainly used in the statistical analysis and to generate images. We used the Kaplan–Meier method to account for differences in the survival rates of the different groups. The ROC curve was used to assess the predicted value of the risk model. The Spearman method was used to calculate the gene expression correlations. The GraphPad Prism (version 9.2, GraphPad Software, La Jolla, CA, USA) was used for statistical analysis of all experimental data. The student’s t-test was performed for between-group comparisons. A *P* value < 0.05 was considered statistically significant.

## Results

### Consensus clustering of m6A methylation regulators identifies 3 subgroups of AML with significant immune relevance

**Fig. 2:**
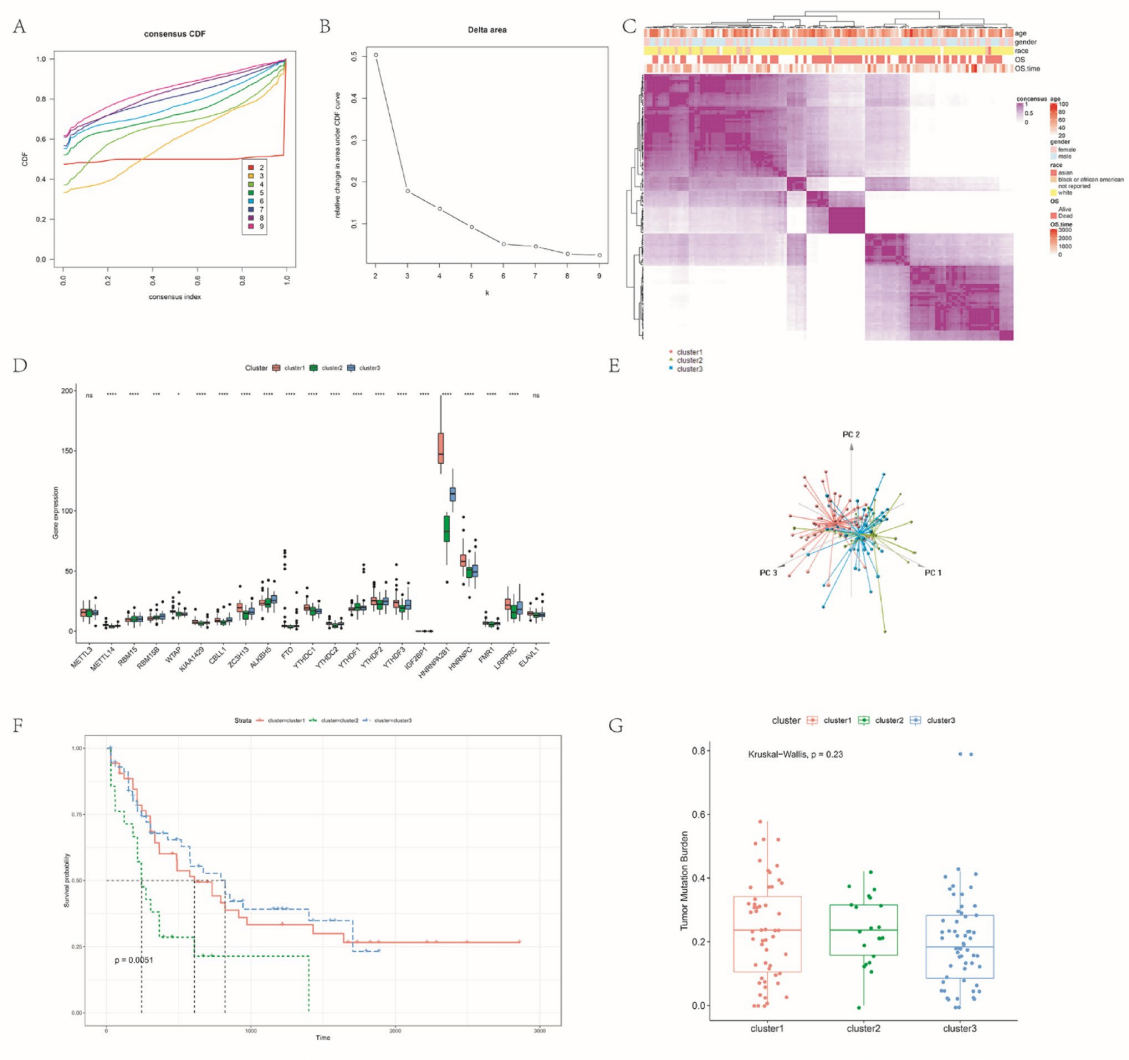
OS of AML in consensus cluster 1/2/3 subgroups identified by m6A RNA methylation regulators. **(A)** Consensus CDF plot. CDF plot of the consensus matrix for each k was shown. **(B)** Delta area plot. This figure shows the relative increase in the clustering consensus following a change from k − 1 to k. **(C)** Heat map. Consensus Clustering Analysis Based on m6A RNA Methylation Regulator Expression in TCGA-AML Cohort Samples. **(D)** Three clusters (cluster 1/2/3) in the violin mapping are defined as consistent expression of m6A RNA methylation regulators. **(E)** Principal component analysis (PCA) of m6A RNA expression profiles. **(F)** Kaplan–Meier overall survival differences in 132 patients with AML. **(G)** TMB box line plot of m6A inter-cluster samples.

The samples of TCGA-AML cohort were consensus clustered according to the expression of 21 m6A RNA methylation regulators. After evaluating the alterations in the cumulative distribution function (CDF) curve and area under the heat map, we clustered the samples into three classes (Fig. 2A–C). We compared the clinical characteristics of these three subgroups clustered by k = 3, namely the subgroups of cluster1, cluster2 and cluster3. We found that 19/21 methylation regulators showed significant differences between two subgroups, and there were also significant differences in expression patterns among different m6A clusters (Fig. 2D, E). In addition, by survival analysis of m6A clusters, we observed that the overall survival of cluster2 subgroup was significantly shorter than that of cluster1 and cluster3 subgroups, and the OS of the three groups was significantly different (*P* = 0.0051) (Fig. 2F). We also calculated the TMB of each sample, however, we found that the TMB of AML patients from the TCGA cohort was less than 1. There was no significant difference observed in the TMB between the different subgroups. (Fig. 2G). We also analysed DEGs among m6A clusters and annotated their functional gene set variation analysis (GSVA) in the biological process. The results showed that DEGs were enriched in immune-related biological processes, including *IL2/STAT5*, interferon response, inflammatory response, etc. (Table S4 and Table 1). These results suggest that the 3 m6A-related AML clusters identified by consensus clustering are closely associated with immune infiltration.

**Table 1:** M6A cluster1 vs cluster3 differential biology pathway.

Biology pathway	logFC	AveExpr	t	*P* value	adj.*P*.Val	B
INTERFERON_GAMMA_RESPONSE	− 0.57314	0.013797	− 9.64146	6.90E−17	1.73E−15	27.76926
MYC_TARGETS_V1	0.493582	− 0.01631	8.670863	1.59E−14	1.99E−13	22.39389
APICAL_JUNCTION	− 0.55437	0.032287	− 8.36162	8.76E−14	7.30E−13	20.70984
MYOGENESIS	− 0.589	0.034042	− 7.79726	1.88E−12	1.18E−11	17.68489
MTORC1_SIGNALING	0.392774	− 0.01722	7.540932	7.42E−12	3.71E−11	16.3352
E2F_TARGETS	0.451805	− 0.01296	6.813695	3.30E−10	1.37E−09	12.60675
ADIPOGENESIS	− 0.36511	0.010089	− 6.71016	5.59E−10	2.00E−09	12.0897
INTERFERON_ALPHA_RESPONSE	− 0.40741	0.009129	− 6.36071	3.22E−09	1.01E−08	10.37336
G2M_CHECKPOINT	0.327673	− 0.01118	6.205556	6.92E−09	1.92E−08	9.626511
HEME_METABOLISM	−0.2622	0.002284	−5.72102	7.06E−08	1.76E−07	7.360194
SPERMATOGENESIS	0.279929	− 0.006	4.380312	2.43E−05	5.53E−05	1.711883
MYC_TARGETS_V2	0.286303	− 0.00472	3.936752	0.000135	0.00028	0.082889
MITOTIC_SPINDLE	− 0.17992	0.015183	− 3.87819	0.000167	0.000321	− 0.12211
APOPTOSIS	− 0.19285	0.003347	− 3.8157	0.00021	0.000375	− 0.33818
PI3K_AKT_MTOR_SIGNALING	− 0.16879	0.007265	− 3.58266	0.000481	0.000802	− 1.11908
PEROXISOME	− 0.16676	0.012492	− 3.38232	0.000953	0.001489	− 1.75812
ALLOGRAFT_REJECTION	− 0.15865	0.007472	− 3.28703	0.001305	0.00192	− 2.05127
UV_RESPONSE_UP	− 0.15331	0.009579	− 3.26856	0.001387	0.001926	− 2.10727
P53_PATHWAY	− 0.14325	0.000757	− 3.03932	0.002873	0.00378	− 2.77996

### Unsupervised clustering of immune genes identified 2 AML subgroups with differentially expressed m6A methylation regulators

**Fig. 3:**
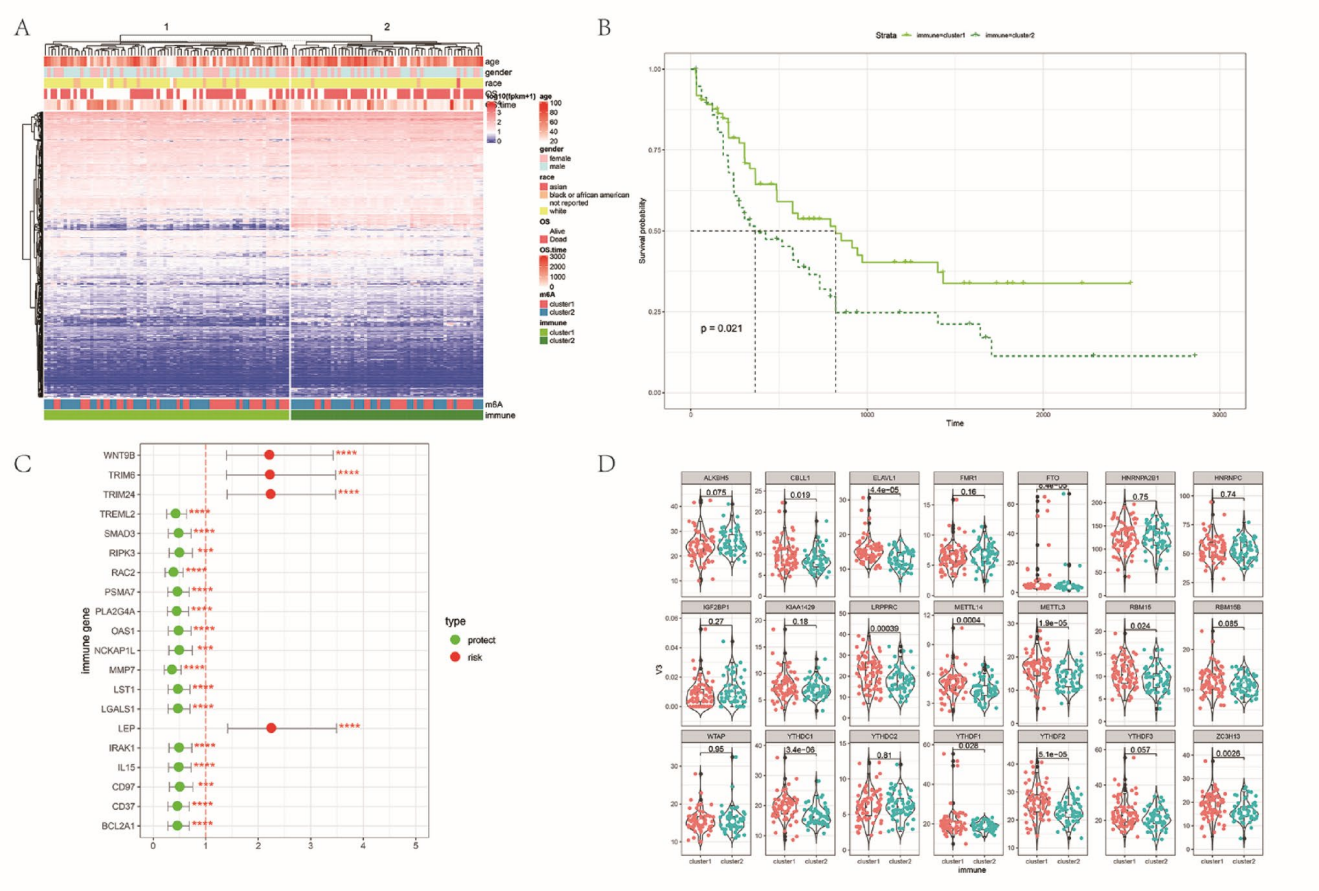
The immune landscape of LAML **(A)** Heat map of immune-associated gene expression. **(B)** Survival analysis among clusters of immune-related genes. **(C)** Odds ratio (OR) values of the top 20 immune genes ranked by P value, hazard ratio (HR), 95% confidence interval (CI) were calculated by Cox regression. **(D)** Differential analysis of 21 m6A methylation regulators among immune-related gene clusters.

By unsupervised cluster analysis, we divided the TCGA-AML samples into 2 clusters (Fig. 3A). The OS was significantly different between immune clusters, and longer for cluster 1 than for cluster 2 (Fig. 3B). By univariate Cox regression analysis, we found 158 immune genes that were significantly associated with prognosis, among which 41 were risk genes, 117 were protective genes (74%). Furthermore, 80% of the top 20 immune genes with significant *P* values were protective genes (Fig. 3C). Analysis of differences in the expression of 21 m6A methylation regulators between clusters of immune-related gene clustering revealed that 11 methylation regulators, including *FTO* (erasers), *CBLL1* (reader), *METTL14* (writer), *ZC3H13* (writer), *METTL3* (writer), *RBM15* (writer), *YTHDC1* (reader), *LRPPRC* (reader), *ELAVL1* (reader), *YTHDF1* (reader) and *YTHDF2* (reader), showed significant differences between immune gene clusters (Fig. 3D). These results showed that different immune-related gene cluster has significantly different expression of m6A methylation regulators.

### WGCNA identifies key m6A-immune-related modules and hub genes

**Fig. 4:**
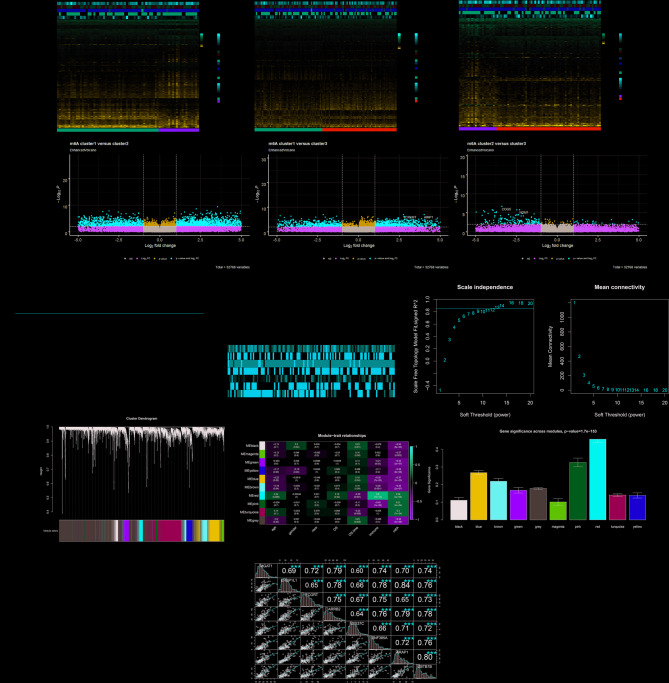
Key modules associated with clinical features identified by WGCNA. **(A, B)** Heat map and volcano map of DEGs between m6A clusters. **(C)** Gene clustering dendrogram: sample outlier display and sample final clustering results. **(D)** Results of soft-thresholding: the relationship between thresholds and features of the network. And the average connectivity in the correlation network constructed for each threshold. **(E)** Dendrogram of all DEGs clustered based on a dissimilarity measure (1-TOM). **(F)** Heat map of correlations between modular features and clinical features: each cell contains correlation coefficients and P values. **(G)** Mean gene significance and error distribution in modules. **(H)** Hub genes show strong correlation.

WGCNA analysis of all DEGs among m6A clusters in TCGA-AML was performed to find the key modules and hub genes that are most associated with m6A methylation and immune infiltration (Fig. 4A, B). The samples with DEGs expression profiles were clustered so as to achieve pruning of the outlier samples with a pruning height of 9000, and then the pruned samples were clustered again. AML sample information such as age, gender, ethnicity, m6A clustering subgroups, immune-related gene clustering subgroups, OS, and survival status was retrieved from the DEGs dataset (Fig. 4C). By setting a soft threshold of 13 (scale-free R2 to 0.85) and a cut height of 0.2, we finally identified 10 key modules (Fig. 4D, E). From the Module-trait relationship heatmap, we found that the red modules have relatively high correlations with clinical features, as well as with m6A and immune infiltration (m6A cluster correlation coefficient = 0.36, *P* = 4E−0.5; immune-related gene cluster correlation coefficient = 0.6, *P* = 1E−13) (Fig. 4F, G). By setting the module membership (MM) > 0.7 and gene significance (GS) > 0.6, we screened 8 hub genes from the red module (*MGAT1, EHBP1L1, FCGRT, ARRB2, VPS37C, ZNF385A, ARAP1,* and *ZBTB7B*). Moreover, as shown in Fig. 4H, we found a significant positive correlation between each of the eight hub genes. This also suggested that these 8 hub genes are closely related to each other.

### Correlation of hub genes with m6A methylation regulators and immune-related genes

**Fig. 5:**
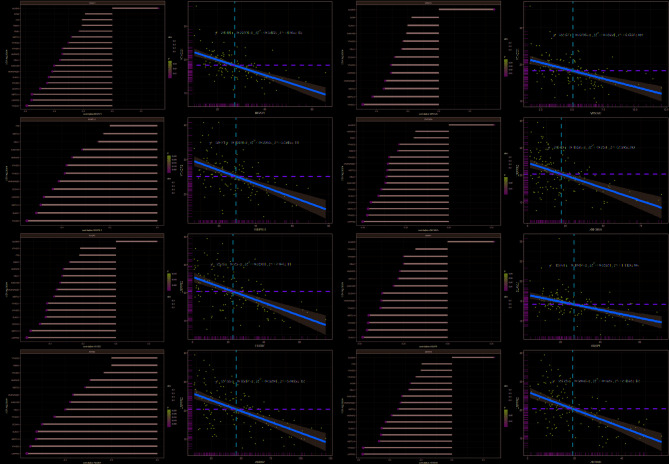
Correlation of hub genes with m6A methylation regulators. Correlation of **(A–H)** hub genes (*MGAT1, EHBP1L1, FCGRT, ARRB2, VPS37C, ZNF385A, ARAP1* with *ZBTB7B*) and m6A methylation regulators, *P* < 0.05.

**Fig. 6:**
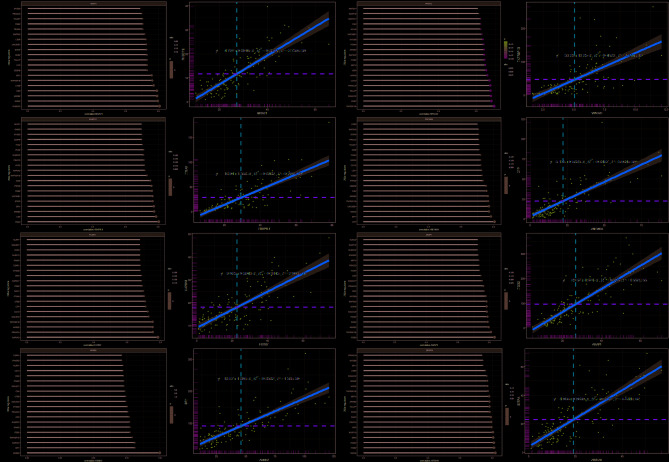
Correlation of hub genes with immune-related genes. Correlation of **(A–H)** hub genes (*MGAT1, EHBP1L1, FCGRT, ARRB2, VPS37C, ZNF385A, ARAP1* with *ZBTB7B*) and m6A methylation regulators, *P* < 0.05.

Correlation analysis revealed that hub gene expression was significantly associated with many m6A RNA methylation regulators. For instance, *MGAT1* was significantly negatively correlated with 17/21 m6A methylation regulators and significantly positively correlated with *ALKBH5*. *EHBP1L1* was significantly negatively correlated with 13/21 m6A methylation regulators. *FCGRT* was significantly negatively correlated with 14/21 m6A methylation regulators and positively correlated with *ALKBH5*. *ARRB2* was negatively significantly correlated with 14/21 m6A methylation regulators. *VPS37C* was significantly negatively correlated with 12/21 m6A methylation regulators and positively correlated with *ALKBH5*. *ZNF385A* was significantly negatively correlated with 15/21 m6A methylation regulators and positively correlated with *ALKBH5*. *ARAP1* was significantly negatively correlated with 13/21 m6A methylation regulators and significantly positively correlated with *ALKBH*. *ZBTB7B* was significantly negatively correlated with 15/21 m6A methylation regulators and positively correlated with *ALKBH5*. These data suggest that hub genes were negatively correlated with most m6A methylation regulators and positively correlated only with *ALKBH5*. In addition, the m6A methylation regulators that are most associated with these hub genes are all methylation-modified regulators (Fig. 5A–H). We then explored potential correlations between the hub genes and the immune-related genes. Interestingly, these 8 hub genes correlate with immune-related genes, just the opposite of m6A methylation regulators. We found that these 8 hub genes were positively correlated with the top 20 immune-related genes, and the correlation was higher than that of m6A methylation regulators (Fig. 6A–H).

### Identification the prognostic value of the hub genes

**Fig. 7:**
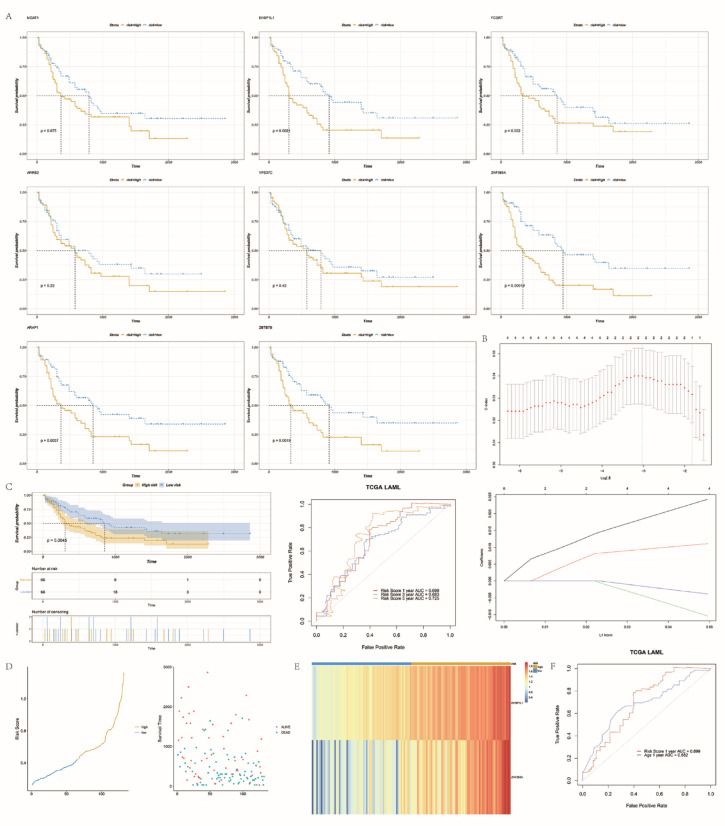
Construction of the risk model. **(A)** Survival analysis of high and low expression groups of 8 hub genes. **(B)** Lasso regression procedure is shown. **(C)** K-M curves, time-dependent ROC curves and LASSO regression analysis of the hub gene risk model high- and low-risk group of TCGA AML patients. **(D)** Survival overview and risk score distribution of the TCGA AML patients. **(E)** Heat map of hub gene risk score in low-risk group and high-risk group. **(F)** One-year AUC of risk score and age.

Regarding prognosis of AML clinical data, Kaplan–Meier curves showed a worse prognosis for high expression of the 8 hub genes. And significant differences between high and low expression groups of hub genes *EHBP1L1* (*P* = 0.0021), *ZNF385A* (*P* = 0.00019), *ARAP1* (*P* = 0.0037), and *ZBTB7B* (*P* = 0.0019) were identified (Fig. 7A). The *EHBP1L1* and *ZNF385A* genes were used to construct the risk model according to the best lambda value of the LASSO regression model (Fig. 7B). Using the coefficients generated by the LASSO regression, we calculated the risk score using the risk score function.

$${\text{Risk score = 0}}{\text{.0126*EHBP1L1}} + {0}{\text{.0068*ZNF385A}}$$

Based on the risk score, patients were divided into low-risk and high-risk groups. We found that the high-risk group had a shorter overall survival and a worse prognosis compared to the low-risk group (*P* = 0.0045, 1-year AUC of 0.699, 3-year AUC of 0.663, and 5-year AUC of 0.725) (Fig. 7C–D). We also found that both hub genes *EHBP1L1* and *ZNF385A* tended to be lowly expressed in the low-risk group and highly expressed in the high-risk group (Fig. 7E). In addition, risk scores had relatively good predictive accuracy for OS compared to age (Fig. 7F).

### Establish a prognostic-prediction model and external validation

**Fig. 8:**
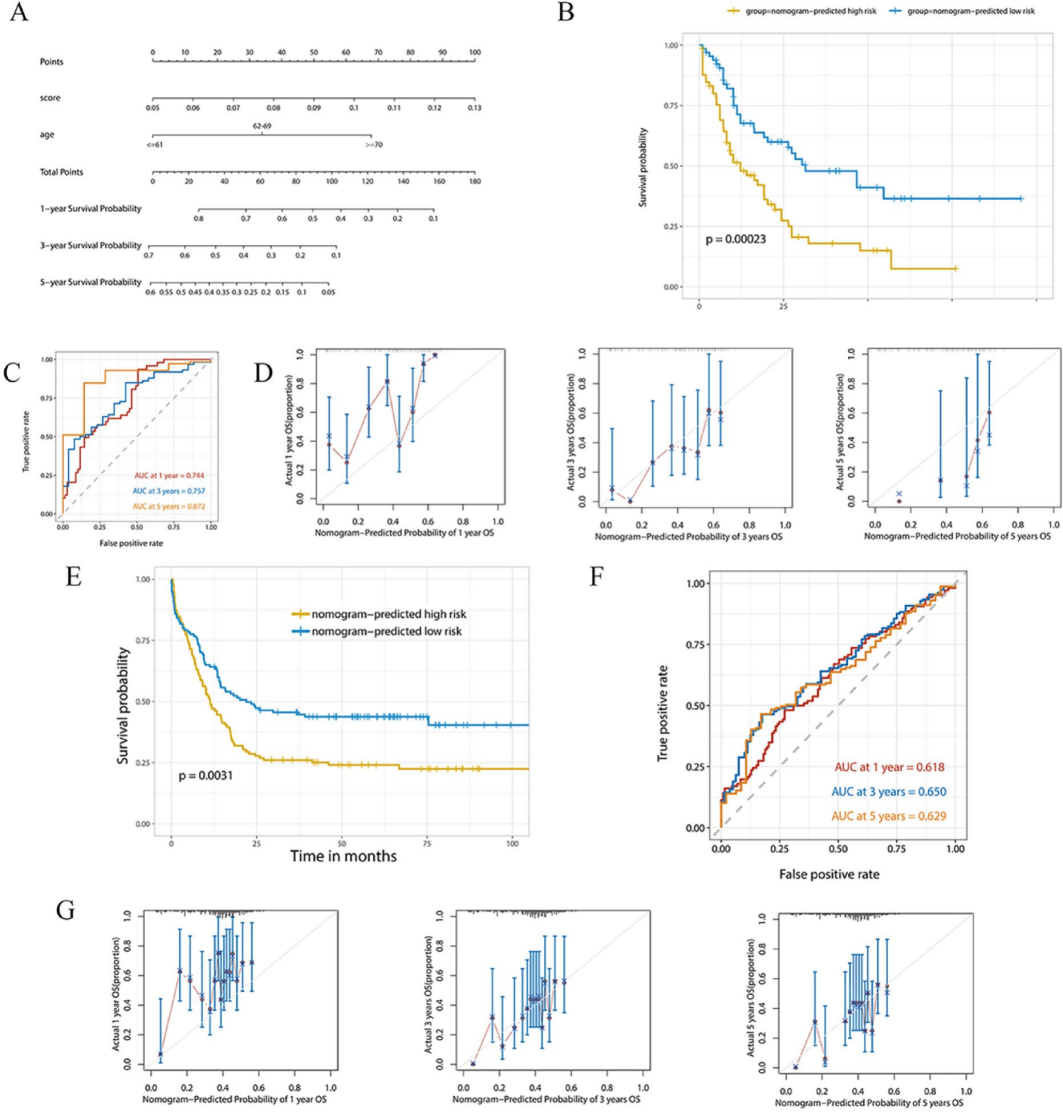
Prognostic analysis of the hub gene and age-base nomogram risk model. **(A)** Hub gene and age-based nomogram prognostic model. **(B)** K-M curves for the nomogram high- and low-risk group of the training set of the TCGA AML patients. **(C)** Time-dependent ROC curves showed the predictive efficiency of hub gene and age-based nomogram prognostic model in the training set of the TCGA AML patients. **(D)** A calibration curve for nomogram risk model in predicting 1/3/5-year survival outcomes for the training set of the TCGA AML patients. **(E)** K-M curves for the nomogram high- and low-risk group of the validation set of the GEO AML patients. **(F)** Time-dependent ROC curves showed the predictive efficiency of hub gene and age-based nomogram prognostic model in the validation set of the GEO AML patients. **(G)** A calibration curve for nomogram risk model in predicting 1/3/5-year survival outcomes for the validation set of the GEO AML patients.

Using the above risk scores, prognostic models were developed based on survival data and RNA expression data from the TCGA AML cohort, combined with clinical variables. One-way Cox regression analysis was performed using the risk score and clinical factors as independent variables in combination with time-to-death outcomes. We found significant differences in hub gene risk scores, age (continuous variable), and age (categorical variable) (*P* < 0.0001). In addition, the hub gene risk score and age (categorical variables) were shown as independent risk factors in the multifactorial COX regression analysis. The multifactorial COX regression model was visualized as a nomogram (Fig. 8A), and the C-index was 0.702. All patients were divided into nomogram-high-risk and nomogram-low-risk groups according to the median value, and a survival curve was created (Fig. 8B). The results suggested that the survival rate in the nomogram-high-risk group was substantially lower than that in the nomogram-low-risk group (median survival was 12.2 months vs. 31.5 months, *P* = 0.00023). The ROC curves for 1/3/5 years indicated that the area under the curve was 0.744, 0.757, and 0.872, respectively, indicating that the prognostic prediction ability of the nomogram is satisfactory, especially at 5 years (Fig. 8C). The 1/3/5-year correction curve for the column line graph shows a better fit for 3 and 5 years, which is consistent with the ROC curve results (Fig. 8D).

We further obtained AML sequencing data GSE23143 at the GEO website for external validation. Combining the hub gene expression and age of each patient of GSE23143, the risk score of death (nomogram-risk score) was calculated for each patient. The results showed a significantly lower survival rate in the high-risk group (*P* = 0.0031, Fig. 8E). The ROC curves for 1/3/5 years indicated that the area under the curve was 0.618, 0.650, and 0.629, respectively (Fig. 8F). The predicted and actual survival rates of the 1/3/5-year calibration curves were generally well-fitted, with the 3-year and 5-year fits being more satisfactory (Fig. 8G). This suggests that the nomogram prediction model has strong predictive capability, especially for long-term forecasts of 3–5 years.

### Hub gene risk score and TME analysis

**Fig. 9:**
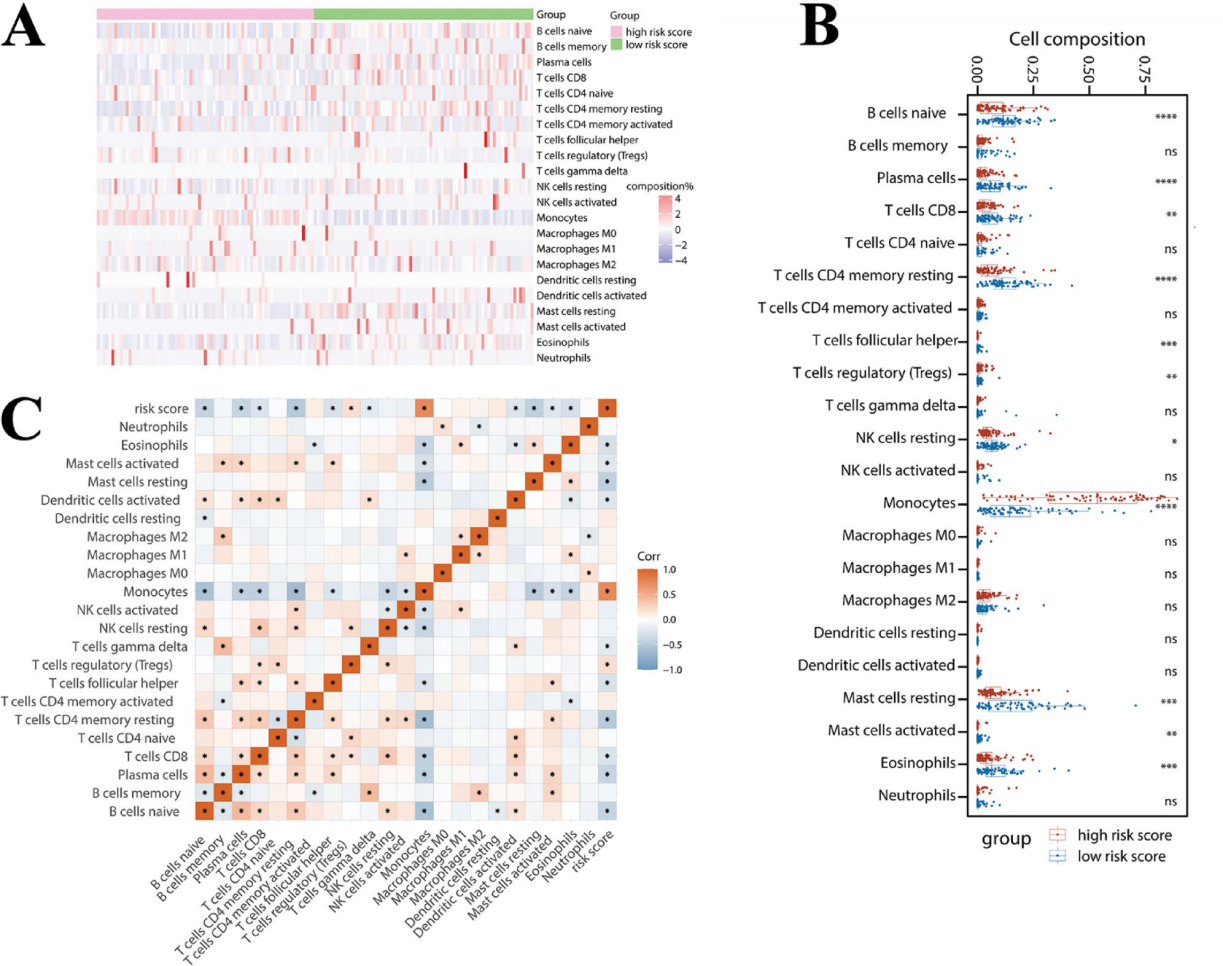
Hub gene risk score and TME analysis in AML patients. **(A)** Heatmap showing the levels of 22 immune cells per patient in the high and low risk groups of the TCGA-LAML cohort. The redder the color, the higher the percentage of cells; the bluer the color the lower the percentage of cells. **(B)** Box line plot showing the levels of 22 immune cells per patient in the high-risk and low-risk groups of the TCGA-LAML. Ns stands for no significance, *P < 0.05; **P < 0.01; ***P < 0.001; ****P < 0.0001. **(C)** Risk scores were analyzed as continuous data for correlation with the proportion of 22 immune cells. Positive correlations are shown in red and negative correlations in blue.

We measured the immune cell composition of TCGA-AML patients in the hub gene score high-risk group and low-risk group by referring to the gene expression signature set of 22 immune cell subtypes provided by the CIBETSORT website. The heat map (Fig. 9A) showed the levels of 22 immune cells for each patient in the high-risk and low-risk groups. There were differences in immune cells in the high- and low-risk groups, such as plasma cells, CD4 memory resting, and monocytes. Further quantification of the box-line and scatter plot (Fig. 9B) suggested that the 11/22 immune cells resulted in significant differences in the high- and low-risk groups. In the results of our analysis, the cell proportions changed the most in monocytes. Using risk score as a continuous variable and analyzing its correlation with each immune cell level, it was found that monocytes and Treg cells were positively correlated with risk score, and T cells CD4 memory resting, plasma cells, B cell naïve, and T cell CD8 were negatively correlated with risk score (Fig. 9C). It suggests that the levels of monocytes and Treg cells are elevated in the high-risk group of patients. This on one hand reflected the ability of the hub gene risk score to distinguish the immune microenvironment characteristics of AML patients with different risk stratification, and on the other hand, further validated the favorable performance of our hub gene risk score.

### Hub gene risk score and immune checkpoints and HLA family genes analysis

**Fig. 10:**
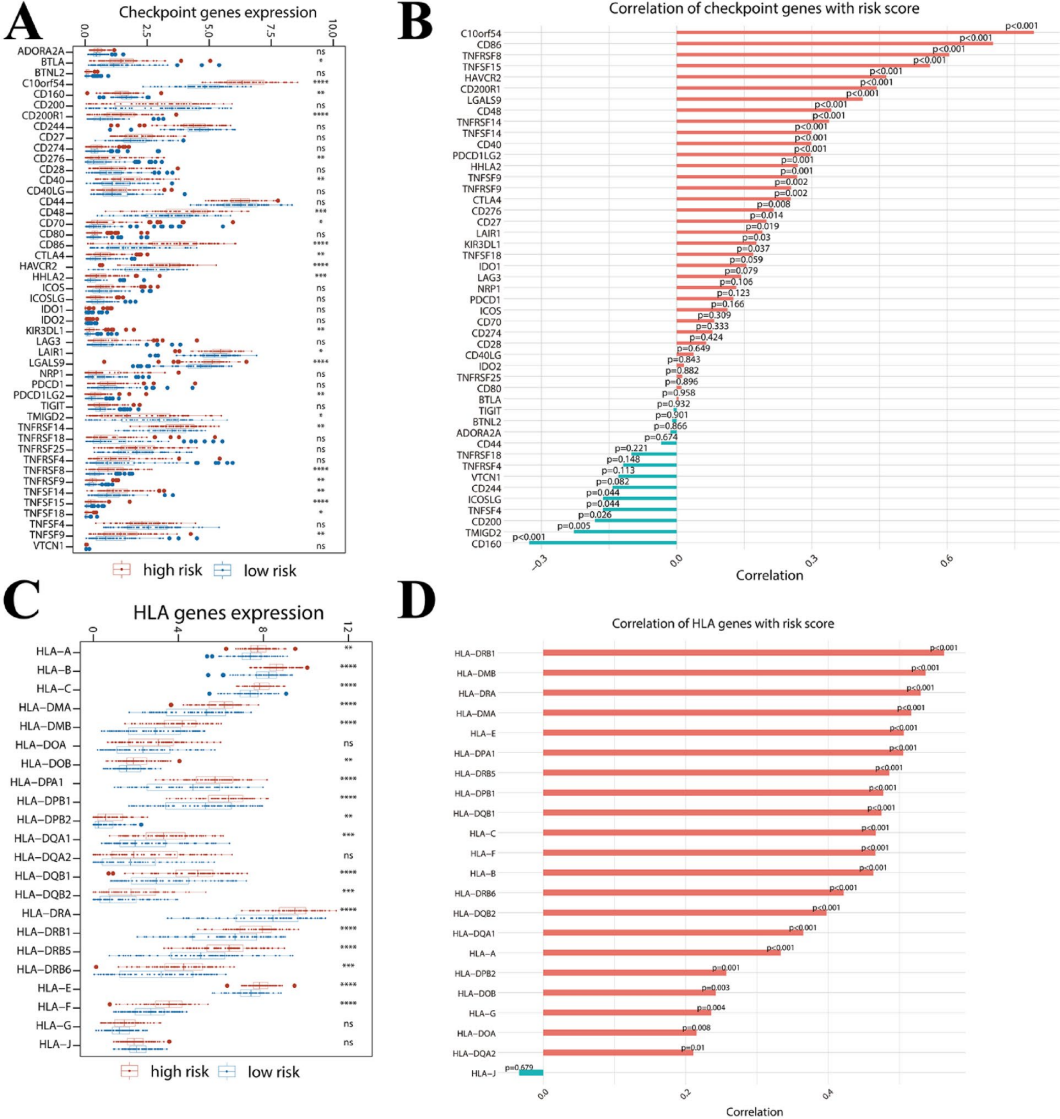
Correlation analysis of risk score with immune checkpoint genes and HLA family genes. **(A)** Differences in immune checkpoint genes between the high- and low- groups. **(B)** Correlation of the risk score as continuous data with the immune checkpoint genes. **(C)** Differences in HLA family genes between the high- and low- groups. **(D)** Correlation of the risk score as continuous data with the HLA family genes. Ns stands for no significance, *P < 0.05; **P < 0.01; ***P < 0.001; ****P < 0.0001. Red bars are positive correlations, blue bars are negative correlations.

Differential expression of immune checkpoint genes and HLA family genes associated with immune characteristics was analyzed in patients in the high-risk and low-risk groups of the hub gene risk score. The results suggested that approximately half of the immune checkpoint genes were significantly different between the two groups and correlated with risk scores (Fig. 10A, B). Genes that were significant and positively correlated with hub gene risk scores included: *VSIR(C10orf54), CD200R1, CD86, HAVCR2 (TIM-3), LGALS9, TNFRSF8, TNFSF15, CD48, HHLA2, CD276 (B7-H3), CD40, CTLA4, TNFRSF14, TNFRSF9, TNFSF14, TNFSF9, LAIR1, TNFSF18,* and *PDCD1LG2 (PD-L2)*. Genes that were significantly and negatively correlated with hub gene risk scores included *CD160* and *TMIGD2*.

The majority of HLA family genes were highly significantly differentially expressed, and were positively correlated with hub gene risk scores (Fig. 10C, D). Genes that were significant and positively correlated with hub gene risk scores included: *HLA-B, HLA-C, HLA-DMA, HLA-DMB, HLA-DPA1, HLA-DPB1, HLA-DQB1, HLA-DRA, HLA-DRB1, HLA-E, HLA-F, HLA-DQA1, HLA-DQB2, HLA-DRB6, HLA-A, HLA-DOB,* and *HLA-DPB2*.

### Hub gene risk score and chemotherapy sensitivity analysis

**Fig. 11:**
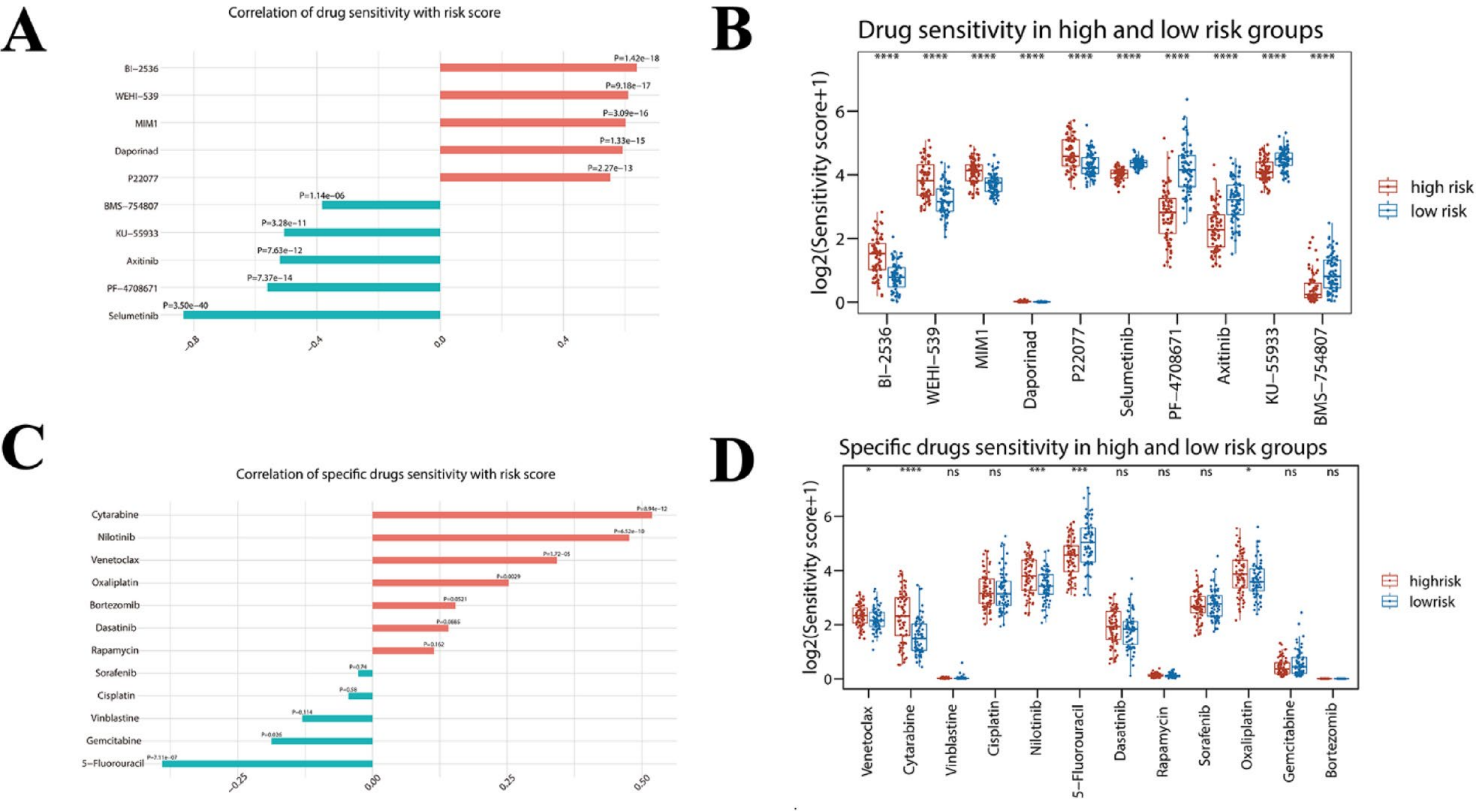
Predictive analysis of drug sensitivity based on hub gene risk score. **(A)** Prediction of sensitivity to 198 chemotherapeutic agents in patients of the TCGA-LAML cohort and screening for the 10 agents with the strongest correlation with risk score (5 positive and 5 negative). **(B)** Box line plot showing the difference in sensitivity of these 10 drugs between patients in the high-risk and low-risk groups. **(C)** The correlation between the sensitivity of 12 clinically used chemotherapeutic and molecularly targeted drugs and the risk score. **(D)** Box plot showing the difference in sensitivity of the 12 drugs between patients in the high-risk and low-risk risk groups. Ns stands for no significance, *P < 0.05; **P < 0.01; ***P < 0.001; ****P < 0.0001.

Based on the “oncoPredict” package in R language, we predicted the top 5 drugs that were most positively and negatively correlated with hub gene risk score (Fig. 11A, B). A positive correlation means that the higher the risk score, the greater the drug sensitivity, which implies that the high-risk group is likely to have a better response to these drugs. According to the analysis (Fig. 11A, B), the top 5 most sensitive drugs in the high-risk group were BI-2536, WEHI-539, MIM1, Daporinad, and P22077. We further screened 12 specific chemotherapeutic agents that widely used in clinical treatment for sensitivity prediction (Fig. 11C, D), and the results showed that 7 of them were positively correlated with risk scores (4 of them were a significant difference). The 4 significantly positively correlated drugs included Cytarabine, Venetoclax, Nilotinib, and Oxaliplatin, which suggested that the high-risk group of hub gene score may respond better to these drugs. Correlation of hub genes with the sensitivity to Cytarabine and Venetoclax was analyzed, and the result showed that *EHBP1L1*, *MGAT1, FCGRT, ARRB2, VPS37C,* and *ARAP1* were significantly positively correlated with both Cytarabine and Venetoclax (Table 2). Based on these results, the risk score may have the potential value to guide clinical decision-making for AML patients in the high-risk group, for whom a high-dose Cytarabine or Venetoclax-based therapy may be clinically feasible.

**Table 2:** Correlation of hub-genes with the sensitivity to Cytarabine and Venetoclax.

Hub-gene	Correlation with cytarabine	*P* value	Correlation with venetoclax	*P* value
EHBP1L1	0.429	3.79E−8	0.297	2.08E−4
MGAT1	0.539	9.34E−13	0.447	9.04E−9
FCGRT	0.525	4.42E−12	0.397	4.47E−7
ARRB2	0.581	5.25E−15	0.283	4.37E−4
VPS37C	0.548	3.33E−13	0.415	1.15E−7
ARAP1	0.483	3.49E−10	0.252	1.79E−3

### Predictive analysis of hub gene risk score and response to immunotherapy

**Fig. 12:**
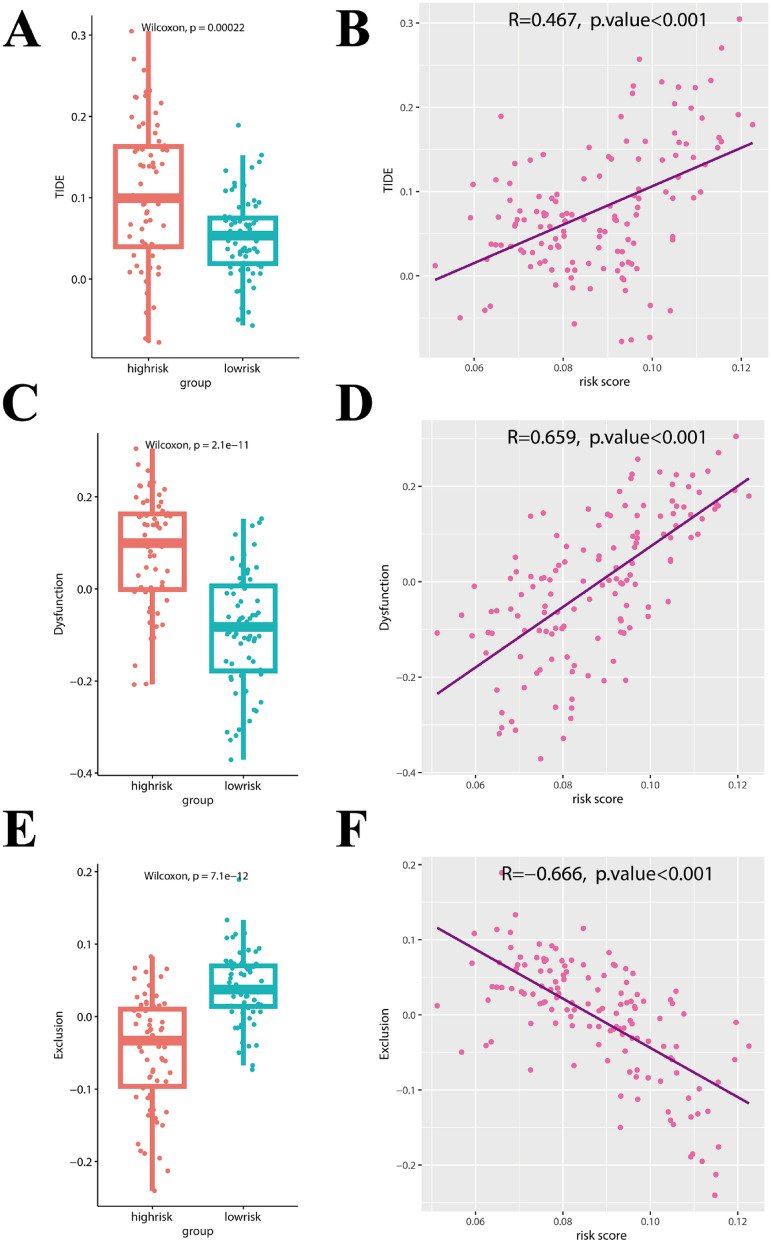
Hub gene risk score and predictive analysis of response to immunotherapy (TIDE analysis). **(A)** Difference between patients’ TIDE scores in the high-risk and low-risk groups and **(B)** correlation with risk scores. **(C)** Difference in patients’ Dysfunction scores between high- and low-risk groups and **(D)** correlation with risk scores. **(E)** Difference in patients’ Exclusion scores between high- and low-risk groups and **(F)** correlation with risk scores.

Tumor immune dysfunction and exclusion (TIDE) includes the two main mechanisms of tumor immune evasion, which are T cell dysfunction and T cell rejection. A higher TIDE prediction score indicates a higher probability of immune escape, suggesting that patients will be less likely to benefit from Immune checkpoint inhibitor (ICI) treatment. The TIDE scores of each patient were counted, and significance comparisons (Fig. 12A) and correlation analysis (Fig. 12B) were performed in the high- and low-risk groups by risk score. In the high-risk group, TIDE scores were significantly higher, which suggested that patients in the high-risk group were less likely to benefit from ICI treatment. This conclusion was supported by correlation analysis, with an extremely strong correlation between the hub gene risk score and the TIDE score (correlation coefficient 0.467, *P* < 0.001).

We further analyzed the relation between hub gene risk score and Dysfunction (T-cell dysfunction) score (Fig. 12C, D) and Exclusion (T-cell rejection) score (Fig. 12E, F). We found that the T-cell dysfunction score in the high-risk group was significantly higher than that in the low-risk group, and the correlation analysis supported the conclusion. Our results showed that in AML patients, with the increase of hub gene risk score, the level of T-cell dysfunction increased, and the level of T-cell rejection decreased. This suggested that the tumor immune evasion ability of AML patients in the high-risk group was mainly derived from T-cell dysfunction rather than T-cell rejection. In the low-risk group, despite the increased T-cell rejection, their overall tumor immune evasion capacity was conversely lower than in the high-risk group due to significantly reduced T-cell dysfunction. Therefore, the low-risk group is more sensitive to ICT treatment and can achieve a better prognosis.

### Verification of hub genes and immune checkpoint genes expression through RT-qPCR

**Fig. 13:**
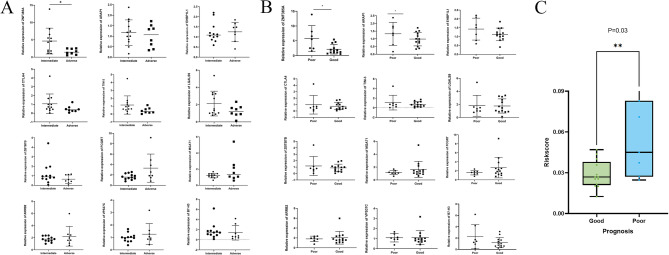
Comprehensive Analysis of m6A Immune-Related Gene Expression and Prognostic Correlations in Clinical Samples of Acute Myeloid Leukemia. **(A)** Analysis of the correlation between the expression of m6A immune-related genes in clinical samples from moderate-risk and high-risk patient groups. **(B)** Difference the level of m6A immune-related between good- and poor-prognosis groups in AML clinical patients. **(C)** RT-qPCR validation and correlation analysis of risk score and prognosis in AML clinical samples.

To verify the differential expression of hub genes and immune checkpoint genes in AML, the total RNA was isolated and performed RT-qPCR. According to the ELN2022 guidelines, all collected AML patients were classified into moderate and high-risk groups. Among these, only the expression of *ZNF385A* exhibited significant differences between the moderate-risk group (n = 12) and high-risk group (n = 8; *P* = 0.03; Fig. 13A).

Patients were further stratified based on their response to induction chemotherapy, dividing them into complete remission (CR/Cri; good prognosis; n = 13) and no response (NR; poor prognosis; n = 7) groups. Results indicated detectable expression levels of 2 hub genes and 8 immune checkpoint genes across all AML patients. Specifically, expression levels of *ZNF385A* and *ARAP1* were notably elevated in the NR group (Fig. 13B). This observation aligns with our experimental validation in AML samples, suggesting that high expression levels of *ZNF385A* may correlate with poor prognosis (*P* = 0.03). However, *EHBP1L1*, utilized in constructing risk models, did not exhibit statistically significant differences between the two groups. It is noteworthy that although the remaining genes (*EHBP1L1*, *ZBTB7B*, *CTLA4, TIM-3, LGALS9*) did not reach statistical significance, there appeared to be a discernible trend.

Additionally, we investigated the relationship between risk scores and prognosis to further validate the accuracy of our risk score model and delineate its clinical relevance. Figure 13C illustrates that the risk score in the good prognosis group was significantly lower than that in the poor prognosis group (*P* = 0.03). This finding holds significant implications for prognostic prediction and personalized treatment strategies in acute myeloid leukemia.

## Discussion

AML is a highly heterogeneous and fatal tumor associated with poor outcomes as a result of the high rate of recurrence^33^. Increasing evidence has demonstrated that m6A modification and its regulatory genes play an essential role in various cancers, including leukemia^34–36^. Although there have been emerging studies in recent years on the role of m6A on TME^24,25,27,28^. However, biomarker models based on m6A-immune-related genes for predicting AML prognosis and drug response have not been reported. Additionally, the effect of m6A modification on AML TME has not been comprehensively understood. Therefore, integrating and analyzing genomic profiles of m6A-immune-related genes from public databases may enhance our understanding of the potential role of m6A regulators and immune signatures in AML and provide more effective treatment strategies.

M6A modification has been demonstrated to be a major post-transcriptional regulator of immune responses in cells^35^. Previous studies showed that a deficiency of *METTL3* impaired mouse T cell homeostasis and differentiation^19,20,37,38^. *YTHDF2* modified transcripts that encode NF-κB, and controlled anti-tumor immunity by regulating intra-tumoral Tregs^14,21^. It is reported that the m6A writer *METTL3* is upregulated during M1 polarization of mouse macrophages by methylating *STAT1* mRNA, which is an important mediator of M1 macrophage polarization^37^. In this study, we determined 3 patterns of m6A regulators and 2 patterns of immune-related genes in AML patients. By survival analysis between m6A clusters, we observed that the OS of the cluster2 subgroup of m6A regulators was significantly shorter. DEGs among m6A clusters were enriched in immune-related biological processes, including *IL2/STAT5*, interferon response, and inflammatory response. Moreover, the OS was significantly different between immune-related gene clusters, and 11 m6A methylation regulators showed significant differences between immune-related gene clusters. These results suggest that the m6A and immune-related genes are mutually affected by each other.

Previous studies have indicated that leukemia stem cells and drug resistance are the main reasons for AML progression and poor outcome^8^. Recent studies showed that both m6A regulators and TME contributed to tumorigenesis, progression, and drug resistance in AML^26–29^. For instance, *FTO* is highly expressed in refractory t (8;21) AML and leads to Ara-C tolerance by targeting *IGFBP2* or lncRNA^9,10^. *ALKBH5* is necessary for the maintenance of AML stem cell function^11,12^. *METTL3* and *YTHDF2* abnormal expression is significantly related to chemoresistance in AML cells^13–15^. In this study, we identified key m6A-immune-related modules and hub genes by WGCNA, and Kaplan–Meier curves indicated poorer outcomes among cases with high expression of the 8 hub genes. Then we constructed a risk model with 2 hub genes (*EHBP1L1*, *P* = 0.0021, and *ZNF385A*, *P* = 0.00019) with the most significant difference by LASSO regression. Based on the risk score, we found that the high-risk group had a shorter OS and a worse prognosis. The hug gene risk score and age were identified as independent risk factors in the multifactorial COX regression analysis in AML. Based on age and risk score, AML patients were further divided into nomogram-high-risk and nomogram-low-risk groups. The results suggest that the survival rate in the nomogram-high-risk group was substantially lower than that in the nomogram-low-risk group (median survival was 12.2 months vs. 31.5 months, *P* = 0.00023). External validation by GSE23143 at the GEO website showed similar results, which suggests that the nomogram prediction model based on m6A-immune-related genes has strong prognostic predictive capability in AML patients, especially for long-term forecasts of 3–5 years.

AML cells employ several mechanisms to establish an immunosuppressive environment to ensure their survival^39^. This is accomplished through the reduction of cytotoxic T and NK cells, increased T cell exhaustion, and recruitment of Tregs and M2 macrophages^40^. The TME was further analyzed according to the hub gene risk score in our study, and we found that monocytes and Treg cells were positively correlated with risk score, and T cells CD4 memory resting, plasma cells, B cell naïve, and T cell CD8 were negatively correlated with risk score. The increase in Tregs and decrease in CD8 + T cells in our study may explain the poor outcome of the high-risk group. Immune checkpoints are also known to be a key mechanism that mediates T cell immunosuppression in AML^41^. T cell exhaustion is often phenotypically characterized by the expression of the immune checkpoint *TIM3*^42–44^. *TIM3* and *CTL4* were significantly correlated with AML relapse and poor OS^42,45^. *Galectin-9 (LGALS9)* regulates immune homeostasis and tumor cell survival through its interaction with its receptor *TIM-3*^46^. Anti-Gal-9 therapy selectively expands intertumoral *TIM-3* + cytotoxic CD8 T cells and immunosuppressive Tregs^44^. *B7-H3* is overexpressed on the leukemic blasts of a significant subset of patients with AML and correlated significantly with a poor outcome^47,48^. Further analysis of differential expression of immune checkpoint genes and HLA family genes in our study showed that a totally of 19 immune checkpoint genes (including *CTLA4, TIM3,* and *LGALS9*) and 17 HLA family genes (including *CD276 (B7-H3)* were significant and positively correlated with hub gene risk scores.

To further investigate the therapy response of AML patients based on m6A-immune-related genes. Specifically, we use the “oncoPredict” package in the R language to predict the top 5 drugs that are most positively and negatively correlated with the hub gene risk score. According to the analysis, the 5 drugs predicted to be most sensitive drugs in the high-risk group were BI-2536, WEHI-539, MIM1, Daporinad, and P22077. We further screened 12 specific chemotherapeutic agents most used in clinical therapy for sensitivity prediction. And the result showed that Cytarabine, Venetoclax, Nilotinib, and Oxaliplatin significantly positively correlated risk score, which means the high-risk group of hub gene score may respond better to these drugs. Cytarabine and Venetoclax are both first-line drugs for the treatment of AML, but patients’ responses can be different^49^. Even the AML patients in the ELN2022 low or moderate groups can experience treatment failure 1. Therefore, it is critical to uncover the genomic properties of AML, and construct prognostic models for predicting prognosis and drug responses. On the basis of our results, the hub gene risk score may have the potential value to guide clinical decision-making for AML patients in the high-risk group, for whom a high-dose cytarabine and Venetoclax-based chemotherapy regimen may be clinically feasible.

ICI has emerged as a therapeutic option in AML for patients who suffer from relapsed or high-risk disease, or patient’s ineligible for standard therapy^43^. To predict ICB response, TIDE was developed, including T cell dysfunction and T cell rejection^50^. A higher TIDE score indicates a higher probability of tumor immune escape and a lower ICI therapy response rate^51^. In our study, we found that based on the hub gene risk score, TIDE scores were significantly higher in the high-risk group. We further analyzed the relation between hub gene risk score and Dysfunction score, and found that T-cell dysfunction score in the high-risk group was significantly higher. Therefore, the low-risk group is expected to be more sensitive to ICT treatment and to have a better prognosis.

To further identify the predictive role of m6A-immune-related genes in AML. We detected the expression of hub genes and immune checkpoint genes using RT-qPCR. The results showed that the expression of *ZNF385A* decreased dramatically in the moderate-risk group when compared with those in the high-risk group, which has statistical significance (*P* = 0.03), and was significantly different between the good and poor prognosis groups. More interesting, *ELHB1L1*, another gene of the risk model, revealed a clear trend, but the risk-score is more meaningful (*P* = 0.03). Therefore, m6A-immune-related genes are highly associated with predicting patient survival, TME, and drug candidates, which may provide insights for individualized therapies and clinical prognosis.

However, this study has certain limitations as follows. First, the sample size of clinical patients in this study was too small. Longer follow-up or larger sample size studies may be needed to reveal potential associations between m6A-immune-related genes and prognosis. Second, the regulation of gene expression in organisms is complex and may be affected by many factors, such as upstream regulatory factors, epigenetic modification, and non-coding RNA. Third, we cannot rule out the possibility that unmeasured factors might contribute to the obtained results. For example, ordinary patients with a limited financial situation cannot afford expensive targeted drugs, which may affect the prognosis of AML patients to a certain extent. Nevertheless, the clear trend of these genes in the prognostic and risk stratification groups is undeniable. Additionally, there was a significant difference between the risk score and prognosis in the samples. Despite these limitations, our study has identified m6A-immune-related genes and successfully established a risk model for predicting prognosis, immune microenvironment, and drug responses. Based on the risk coefficient assessment, clinicians can better understand the patient’s disease status, thereby developing a more accurate treatment plan.

In conclusion, from the TCGA database and WGCNA analysis, m6A-immune-related genes were identified and a risk score model was constructed with robust prognostic value. This model can predict prognosis, immune microenvironment, and drug responses in AML patients.

## Supplementary Information

Below is the link to the electronic supplementary material.

Supplementary Material 1

## Data Availability

The datasets presented in this study can be found in online repositories. The names of the repository/repositories and accession number(s) can be found below: TCGA https://portal.gdc.cancer.gov/ ; UCSC https://xenabrowser.net/datapages/ ; GEO https://www.ncbi.nlm.nih.gov/geo/ ; GSE23143 https://xenabrowser.net/datapages/ ; M Sig DB database https://www.gsea-msigdb.org/gsea/msigdb ; Innate DB database https://www.innatedb.ca/ ; CIBERSORTx http://cibersortx.stanford.edu/ ; TIDE http://tide.dfci.harvard.edu/

## References

[1] Pollyea, D. A. et al. Acute myeloid leukemia, Version 3.2023, NCCN clinical practice guidelines in oncology. *J. Natl. Compr. Canc. Netw.* **21**(5), 503–513. 10.6004/jnccn.2023.0025 (2023). 37156478 10.6004/jnccn.2023.0025

[2] Li, D. et al. Identification of m6A-related lncRNAs associated with prognoses and immune responses in acute myeloid leukemia. *Front. Cell Dev. Biol.*10.3389/fcell.2021.770451 (2021). 34869365 10.3389/fcell.2021.770451 PMC8637120

[3] Yeung, Y. A. et al. An optimized full-length FLT3/CD3 bispecific antibody demonstrates potent anti-leukemia activity and reversible hematological toxicity. *Mol. Ther.* **28**(3), 889–900. 10.1016/j.ymthe.2019.12.014 (2020). 31981494 10.1016/j.ymthe.2019.12.014 PMC7054815

[4] Zheng, X. & Gong, Y. Functions of RNA N6-methyladenosine modification in acute myeloid leukemia. *Biomark. Res.*10.1186/s40364-021-00293-w (2021). 34001273 10.1186/s40364-021-00293-w PMC8130309

[5] Liu, K. et al. Pharmacoepitranscriptomic landscape revealing m6A modification could be a drug-effect biomarker for cancer treatment. *Mol. Ther. Nucleic Acids*10.1016/j.omtn.2022.04.001 (2022). 35505958 10.1016/j.omtn.2022.04.001 PMC9044172

[6] Zhao, Y. et al. m6A-dependent upregulation of DDX21 by super-enhancer-driven IGF2BP2 and IGF2BP3 facilitates progression of acute myeloid leukaemia. *Clin. Transl. Med.* **14**(4), e1628. 10.1002/ctm2.1628 (2024). 38572589 10.1002/ctm2.1628 PMC10993053

[7] Feng, G. et al. Small molecule inhibitors targeting m6A regulators. *J. Hematol. Oncol.* **17**(1), 30. 10.1186/s13045-024-01546-5 (2024). 38711100 10.1186/s13045-024-01546-5 PMC11075261

[8] Li, Z. et al. Integrated analysis of single-cell RNA-seq and bulk RNA-seq reveals RNA N6-methyladenosine modification associated with prognosis and drug resistance in acute myeloid leukemia. *Front. Immunol.* **14**, 1281687. 10.3389/fimmu.2023.1281687 (2023). 38022588 10.3389/fimmu.2023.1281687 PMC10644381

[9] Zhou, W. et al. A novel AML1-ETO/FTO positive feedback loop promotes leukemogenesis and Ara-C resistance via stabilizing IGFBP2 in t(8;21) acute myeloid leukemia. *Exp. Hematol. Oncol.*10.1186/s40164-024-00480-z (2024). 38268050 10.1186/s40164-024-00480-z PMC10807068

[10] Kou, R. et al. Exosome-shuttled FTO from BM-MSCs contributes to cancer malignancy and chemoresistance in acute myeloid leukemia by inducing m6A-demethylation: A nano-based investigation. *Environ. Res.* **244**, 117783. 10.1016/j.envres.2023.117783 (2024). 38048862 10.1016/j.envres.2023.117783

[11] Li, R. et al. RNA demethylase ALKBH5 promotes tumorigenesis of t (8;21) acute myeloid leukemia via ITPA m6A modification. *Biomark. Res.* **11**(1), 30. 10.1186/s40364-023-00464-x (2023). 36899424 10.1186/s40364-023-00464-x PMC10007764

[12] Wang, P. et al. Deubiquitinase USP9X stabilizes RNA m6A demethylase ALKBH5 and promotes acute myeloid leukemia cell survival. *J. Biol. Chem.* **299**(8), 105055. 10.1016/j.jbc.2023.105055 (2023). 37454738 10.1016/j.jbc.2023.105055 PMC10424212

[13] Liao, X. et al. Deletion of Mettl3 in mesenchymal stem cells promotes acute myeloid leukemia resistance to chemotherapy. *Cell. Death Dis.* **14**(12), 796. 10.1038/s41419-023-06325-7 (2023). 38052820 10.1038/s41419-023-06325-7 PMC10698052

[14] Zhang, Z. et al. RNA m6A reader YTHDF2 facilitates precursor miR-126 maturation to promote acute myeloid leukemia progression. *Genes Dis.* **11**(1), 382–396. 10.1016/j.gendis.2023.01.016 (2024). 37588203 10.1016/j.gendis.2023.01.016 PMC10425806

[15] Wu, C., Cui, J., Huo, Y., Shi, L. & Wang, C. Alternative splicing of HOXB-AS3 underlie the promoting effect of nuclear m6A reader YTHDC1 on the self-renewal of leukemic stem cells in acute myeloid leukemia. *Int. J. Biol. Macromol.* **237**, 123990. 10.1016/j.ijbiomac.2023.123990 (2023). 36906205 10.1016/j.ijbiomac.2023.123990

[16] Shulman, Z. & Stern-Ginossar, N. The RNA modification N6-methyladenosine as a novel regulator of the immune system. *Nat. Immunol.* **21**(5), 501–512. 10.1038/s41590-020-0650-4 (2020). 32284591 10.1038/s41590-020-0650-4

[17] Wang, Y.-N., Yu, C.-Y. & Jin, H.-Z. RNA N6-methyladenosine modifications and the immune response. *J. Immunol. Res.* **2020**, 6327614. 10.1155/2020/6327614 (2020). 32411802 10.1155/2020/6327614 PMC7204177

[18] Ouyang, X. & Gong, Y. One stone, two birds: N6-methyladenosine RNA Modification in leukemia stem cells and the tumor immune microenvironment in acute myeloid leukemia. *Front. Immunol.* **13**, 912526. 10.3389/fimmu.2022.912526 (2022). 35720276 10.3389/fimmu.2022.912526 PMC9201081

[19] Su, W., Che, L., Liao, W. & Huang, H. The RNA m6A writer METTL3 in tumor microenvironment: Emerging roles and therapeutic implications. *Front. Immunol.* **15**, 1335774. 10.3389/fimmu.2024.1335774 (2024). 38322265 10.3389/fimmu.2024.1335774 PMC10845340

[20] Wang, Z. et al. Suppression of the METTL3-m6A-integrin β1 axis by extracellular acidification impairs T cell infiltration and antitumor activity. *Cell. Rep.* **43**(2), 113796. 10.1016/j.celrep.2024.113796 (2024). 38367240 10.1016/j.celrep.2024.113796

[21] Zhang, L. et al. YTHDF2/m6 A/NF-κB axis controls anti-tumor immunity by regulating intratumoral tregs. *EMBO J.* **42**(15), e113126. 10.15252/embj.2022113126 (2023). 37345898 10.15252/embj.2022113126 PMC10390869

[22] Hong, Y.-G. et al. The RNA m6A reader YTHDF1 is required for acute myeloid leukemia progression. *Cancer Res.* **83**(6), 845–860. 10.1158/0008-5472.CAN-21-4249 (2023). 36634204 10.1158/0008-5472.CAN-21-4249

[23] Zálešák, F. et al. Structure-based design of a potent and selective YTHDC1 ligand. *J. Med. Chem.*10.1021/acs.jmedchem.4c00599 (2024). 38787793 10.1021/acs.jmedchem.4c00599 PMC11181329

[24] Li, J., Zhang, H. & Wang, H. N1-methyladenosine modification in cancer biology: Current status and future perspectives. *Comput. Struct. Biotechnol. J.* **20**, 6578–6585. 10.1016/j.csbj.2022.11.045 (2022). 36467585 10.1016/j.csbj.2022.11.045 PMC9712505

[25] Liao, X. et al. m6A RNA methylation regulators predict prognosis and indicate characteristics of tumour microenvironment infiltration in acute myeloid leukaemia. *Epigenetics*10.1080/15592294.2022.2160134 (2023). 36567510 10.1080/15592294.2022.2160134 PMC9980463

[26] Yuan, S. et al. Analysis of m6A-related signatures associated with the tumor immune microenvironment and predict survival in acute myeloid leukemia. *Ann. Transl. Med.* **10**(16), 902. 10.21037/atm-22-3858 (2022). 36111007 10.21037/atm-22-3858 PMC9469131

[27] Xu, Z.-J. et al. m6A regulator-based methylation modification patterns and characterization of tumor microenvironment in acute myeloid leukemia. *Front. Genet.* **13**, 948079. 10.3389/fgene.2022.948079 (2022). 36035161 10.3389/fgene.2022.948079 PMC9399688

[28] Han, S. et al. Characterization of m6A regulator-mediated methylation modification patterns and tumor microenvironment infiltration in acute myeloid leukemia. *Cancer Med.* **11**(5), 1413–1426. 10.1002/cam4.4531 (2022). 35023630 10.1002/cam4.4531 PMC8894699

[29] Du, A. et al. m6A regulator-mediated methylation modification patterns and tumor microenvironment infiltration characterization in acute myeloid leukemia. *Front. Immunol.* **12**, 789914. 10.3389/fimmu.2021.789914 (2021). 34887874 10.3389/fimmu.2021.789914 PMC8650218

[30] Zhang, B. et al. m6A regulator-mediated methylation modification patterns and tumor microenvironment infiltration characterization in gastric cancer. *Mol. Cancer* **19**(1), 53. 10.1186/s12943-020-01170-0 (2020). 32164750 10.1186/s12943-020-01170-0 PMC7066851

[31] Chen, B., Khodadoust, M. S., Liu, C. L., Newman, A. M. & Alizadeh, A. A. Profiling tumor infiltrating immune cells with CIBERSORT. *Methods Mol. Biol.* **1711**, 243–259. 10.1007/978-1-4939-7493-1_12 (2018). 29344893 10.1007/978-1-4939-7493-1_12 PMC5895181

[32] Ju, M. et al. Pan-cancer analysis of NLRP3 inflammasome with potential implications in prognosis and immunotherapy in human cancer. *Br. Bioinform.* **22**(4), bba345. 10.1093/bib/bbaa345 (2021). 10.1093/bib/bbaa345 PMC8294515 33212483

[33] DiNardo, C. D., Erba, H. P., Freeman, S. D. & Wei, A. H. Acute myeloid leukaemia. *Lancet* **401**(10393), 2073–2086. 10.1016/S0140-6736(23)00108-3 (2023). 37068505 10.1016/S0140-6736(23)00108-3

[34] Deng, L.-J. et al. m6A modification: Recent advances, anticancer targeted drug discovery and beyond. *Mol. Cancer* **21**(1), 52. 10.1186/s12943-022-01510-2 (2022). 35164788 10.1186/s12943-022-01510-2 PMC8842557

[35] Yue, S.-W. et al. m6A-regulated tumor glycolysis: New advances in epigenetics and metabolism. *Mol. Cancer* **22**(1), 137. 10.1186/s12943-023-01841-8 (2023). 37582735 10.1186/s12943-023-01841-8 PMC10426175

[36] Chen, X.-Y., Zhang, J. & Zhu, J.-S. The role of m6A RNA methylation in human cancer. *Mol. Cancer.* **18**(1), 103. 10.1186/s12943-019-1033-z (2019). 31142332 10.1186/s12943-019-1033-z PMC6540575

[37] Liu, Y. et al. The N6-methyladenosine (m6A)-forming enzyme METTL3 facilitates M1 macrophage polarization through the methylation of STAT1 mRNA. *Am. J. Physiol. Cell. Physiol.* **317**(4), C762–C775. 10.1152/ajpcell.00212.2019 (2019). 31365297 10.1152/ajpcell.00212.2019

[38] Liu, C. et al. Potential roles of N6-methyladenosine (m6A) in immune cells. *J. Transl. Med.* **19**(1), 251. 10.1186/s12967-021-02918-y (2021). 34103054 10.1186/s12967-021-02918-y PMC8186046

[39] Gurska, L. & Gritsman, K. Unveiling T cell evasion mechanisms to immune checkpoint inhibitors in acute myeloid leukemia. *Cancer Drug Resist.* **6**(3), 674–687. 10.20517/cdr.2023.39 (2023). 37842238 10.20517/cdr.2023.39 PMC10571054

[40] Vago, L. & Gojo, I. Immune escape and immunotherapy of acute myeloid leukemia. *J. Clin. Invest.* **130**(4), 1552–1564. 10.1172/JCI129204 (2020). 32235097 10.1172/JCI129204 PMC7108895

[41] Bakhtiyari, M. et al. The role of bone marrow microenvironment (BMM) cells in acute myeloid leukemia (AML) progression: Immune checkpoints, metabolic checkpoints, and signaling pathways. *Cell Commun. Signal.* **21**(1), 252. 10.1186/s12964-023-01282-2 (2023). 37735675 10.1186/s12964-023-01282-2 PMC10512514

[42] Kong, Y. et al. PD-1(hi)TIM-3(+) T cells associate with and predict leukemia relapse in AML patients post allogeneic stem cell transplantation. *Blood Cancer J.* **5**(7), e330. 10.1038/bcj.2015.58 (2015). 26230954 10.1038/bcj.2015.58 PMC4526784

[43] Gómez-Llobell, M., Peleteiro Raíndo, A., Climent Medina, J., Gómez Centurión, I. & Mosquera, O. A. Immune checkpoint inhibitors in acute myeloid leukemia: A meta-analysis. *Front. Oncol.* **12**, 882531. 10.3389/fonc.2022.882531 (2022). 35530329 10.3389/fonc.2022.882531 PMC9069679

[44] Yang, R. et al. Galectin-9 interacts with PD-1 and TIM-3 to regulate T cell death and is a target for cancer immunotherapy. *Nat. Commun.* **12**(1), 832. 10.1038/s41467-021-21099-2 (2021). 33547304 10.1038/s41467-021-21099-2 PMC7864927

[45] Chen, C. et al. Expression patterns of immune checkpoints in acute myeloid leukemia. *J. Hematol. Oncol.* **13**(1), 28. 10.1186/s13045-020-00853-x (2020). 32245463 10.1186/s13045-020-00853-x PMC7118887

[46] Lv, Y., Ma, X., Ma, Y., Du, Y. & Feng, J. A new emerging target in cancer immunotherapy: Galectin-9 (LGALS9). *Genes Dis.* **10**(6), 2366–2382. 10.1016/j.gendis.2022.05.020 (2023). 37554219 10.1016/j.gendis.2022.05.020 PMC10404877

[47] Lichtman, E. I. et al. Preclinical evaluation of B7–H3-specific chimeric antigen receptor T cells for the treatment of acute myeloid leukemia. *Clin. Cancer Res.* **27**(11), 3141–3153. 10.1158/1078-0432.CCR-20-2540 (2021). 33531429 10.1158/1078-0432.CCR-20-2540 PMC8248479

[48] Zhang, L.-Y. et al. Integrated analysis reveals distinct molecular, clinical, and immunological features of B7–H3 in acute myeloid leukemia. *Cancer Med.* **10**(21), 7831–7846. 10.1002/cam4.4284 (2021). 34562306 10.1002/cam4.4284 PMC8559480

[49] Wei, A. H. et al. Venetoclax plus LDAC for newly diagnosed AML ineligible for intensive chemotherapy: A phase 3 randomized placebo-controlled trial. *Blood* **135**(24), 2137–2145. 10.1182/blood.2020004856 (2020). 32219442 10.1182/blood.2020004856 PMC7290090

[50] Jiang, P. et al. Signatures of T cell dysfunction and exclusion predict cancer immunotherapy response. *Nat. Med.* **24**(10), 1550–1558. 10.1038/s41591-018-0136-1 (2018). 30127393 10.1038/s41591-018-0136-1 PMC6487502

[51] Feng, S. et al. Immunogenic cell death related risk model to delineate ferroptosis pathway and predict immunotherapy response of patients with GBM. *Front. Immunol.* **13**, 992855. 10.3389/fimmu.2022.992855 (2022). 36248827 10.3389/fimmu.2022.992855 PMC9554879

