# Supplementary material for: Identification of a m6A-immune-related risk model for predicting prognosis, immune microenvironment, and drug responses in acute myeloid leukemia
Source: Sci Rep. 2025 Nov 3;15:38306. doi: 10.1038/s41598-025-22002-5 (PMC12583824; doi:10.1038/s41598-025-22002-5)
Supplement: Supplementary file 1 — Supplementary Material 1 [file 41598_2025_22002_MOESM1_ESM.zip › Supplementary_Material/Table S1.docx]

**Table S1.** Details on AML patient samples by RT-qPCR.

| NO. | Gender | Age | | WBC  (×10^9/L) | | Chromosome | | Mutant genes  (Grade I mutation) | ELN2022 | | Prognosis | | |
| --- | --- | --- | --- | --- | --- | --- | --- | --- | --- | --- | --- | --- | --- |
| AML#1 | Male | 47 | 17.41 | | 46, XY, inv(16)(p13; q22)[15] | | FLT3-TKD | | | Intermediate | | NR |  |
| AML#2 | Male | 75 | 1.61 | | 46, XX[10] | | TP53; SF3B1; DNMT3A | | | Adverse | | NR |  |
| AML#3 | Female | 78 | 188.99 | | No metaphase cells observed | | NPM1; IDH1; FLT3-TKD; PTPN11; FLT3-ITD | | | Intermediate | | NR |  |
| AML#4 | Female | 68 | 79.86 | | 46, XX[8] | | TET2; NPM1; FLT3-ITD | | | Intermediate | | NR |  |
| AML#5 | Male | 63 | 171.1 | | 45, X, -Y[4] | | NPM1; DNMT3A; FLT3-ITD | | | Intermediate | | NR |  |
| AML#6 | Female | 72 | 41.43 | | 46, XX[20] | | SF3B1; NRAS; FLT3-ITD | | | Adverse | | NR |  |
| AML#7 | Male | 56 | 16.62 | | 46, XY, t(8; 21)(q22; q22)[10]/46,XY[10] | | NRAS; ASXL2 | | | Intermediate | | NR |  |
| AML#8 | Male | 41 | 52.9 | | 46, XY, t(8; 21)(q22; q22)[20] | | ASXL2; NRAS; CCND2 | | | Intermediate | | NR |  |
| AML#9 | Female | 67 | 47.51 | | None | | DNMT3A; IDH1; NPM1; FLT3-ITD | | | Intermediate | | CR/CRi |  |
| AML#10 | Female | 55 | 6.17 | | 46, XX[20] | | CEBPA | | | Intermediate | | CR/CRi |  |
| AML#11 | Female | 60 | 143.97 | | 46, XX[20] | | NOTCH1; IDH2; NPM1; CEBPA; FLT3-ITD | | | Intermediate | | CR/CRi |  |
| AML#12 | Female | 17 | 347.26 | | 46, XX, del(4)(p15; p11)[12] | | SRSF2; NRAS; WT1 | | | Adverse | | CR/CRi |  |
| AML#13 | Female | 50 | 6.69 | | 46, XX[20] | | DNMT3A; NPM1; NF1 | | | Adverse | | CR/CRi |  |
| AML#14 | Male | 51 | 5.41 | | None | | CEBPA; TET2 | | | Intermediate | | CR/CRi |  |
| AML#15 | Male | 19 | 26.88 | | 46, XY, t(8; 21)(q22; q22)[20] | | JAK2 | | | Intermediate | | CR/CRi |  |
| AML#16 | Male | 19 | 22.57 | | 46, XY,t(5; 6)(q33; q23),-7,t(9; 22)(q34; q11)[20] | | SETD2 | | | Adverse | | CR/CRi |  |
| AML#17 | Male | 26 | 1.56 | | 47, XY, +4[5]/46, XY[30] | | IDH2; NPM1 | | | Intermediate | | CR/CRi |  |
| AML#18 | Female | 87 | 118.85 | | 46, XY[20] | | TET2; SRSF2; NPM1; FLT3 | | | Adverse | | CR/CRi |  |
| AML#19 | Male | 86 | 2.98 | | 45, X, -Y[9]/46, XY[18] | | ZRSR2; CEBPA; TET2; PHF6 | | | Adverse | | CR/CRi |  |
| AML#20 | Female | 59 | 66.19 | | 46, XX[20] | | NPM1; FLT3-TKD | | | Adverse | | CR/CRi |  |
