# Supplementary material for: Identification of a m6A-immune-related risk model for predicting prognosis, immune microenvironment, and drug responses in acute myeloid leukemia
Source: Sci Rep. 2025 Nov 3;15:38306. doi: 10.1038/s41598-025-22002-5 (PMC12583824; doi:10.1038/s41598-025-22002-5)
Supplement: Supplementary file 1 — Supplementary Material 1 [file 41598_2025_22002_MOESM1_ESM.zip › Supplementary_Material/Table S2.docx]

**Table S2.** The clinical characteristics for AML cases in TCGA.

| Characteristics | High risk group  N=66 | Low risk group  N=66 | P value  of difference |
| --- | --- | --- | --- |
| Median age, year (range) | 60.5 (21, 88) | 51.2 (21, 81) | 0.007 |
| Sex |  |  | 0.861 |
| Female, n (%) | 31 (47%) | 30 (45.5%) |  |
| Male, n (%) | 35 (53%) | 36 (53.8%) |  |
| FLT3 |  |  | 0.575 |
| Negative, n (%) | 45 (68.2%) | 45 (68.2%) |  |
| Positive, n (%) | 20 (30.3%) | 18 (27.3%) |  |
| NA, n (%) | 1 (1.5%) | 3 (4.5%) |  |
| IDH1 |  |  | 0.099 |
| Negative, n (%) | 56 (84.8%) | 48 (72.7%) |  |
| Positive, n (%) | 10 (15.2%) | 15 (22.7%) |  |
| NA, n (%) | 0 (0%) | 3 (4.5%) |  |
| RAS |  |  | 0.47 |
| Negative, n (%) | 61 (92.4%) | 62 (93.9%) |  |
| Positive, n (%) | 5 (7.6%) | 3 (4.5%) |  |
| NA, n (%) | 0 (0%) | 1 (1.5%) |  |
| NPM1 |  |  | 0.23 |
| Negative, n (%) | 47 (71.2%) | 53 (80.3%) |  |
| Positive, n (%) | 19 (28.8%) | 12 (18.2%) |  |
| NA, n (%) | 0 (0%) | 1 (1.5%) |  |
