# Supplementary material for: Identification of a m6A-immune-related risk model for predicting prognosis, immune microenvironment, and drug responses in acute myeloid leukemia
Source: Sci Rep. 2025 Nov 3;15:38306. doi: 10.1038/s41598-025-22002-5 (PMC12583824; doi:10.1038/s41598-025-22002-5)
Supplement: Supplementary file 1 — Supplementary Material 1 [file 41598_2025_22002_MOESM1_ESM.zip › Supplementary_Material/Table S3.docx]

**Table S3.** The list for sequences of primers used for RT-qPCR.

| Name of the amplification primer | Enlarge the sequence of primers |
| --- | --- |
| β-actin-F | GTCCACCGCAAATGCTTCTA |
| β-actin-R | TGCTGTCACCTTCACCGTTC |
| H-ZNF385A F | TGCTCTACTGTGCTCTGTGC |
| H-ZNF385A R | ACTTCGGGCCTCCAGAATTG |
| H-ARAP1 F | GGGCCCCTGAGACCAG |
| H-ARAP1 R | GGGGATCAGTGACACACTACC |
| H-ZBTB7B F | TGCACAGCTACGACCTCAAG |
| H-ZBTB7B R | GGTAGAGGGTGGTGGGTAGT |
| H-B7-H3 F | GTGGTTCTGCCTCACAGGAG |
| H-B7-H3 R | GCCAGATGAGGTTGAGCTGT |
| H-CTLA4 F | GAAGTCTGTGCGGCAACCTA |
| H-CTLA4 R | TGGCCCTCAGTCCTTGGATA |
| H-TIM-3 F | GGAATACAGAGCGGAGGTCG |
| H-TIM-3 R | GGACACATCTCCTTTGCGGA |
| H-LGALS9 F | CCCGAGGAGAGGAAGACACA |
| H-LGALS9 R | TCCCGTTCACCATCACCTTG |
| H-DNMT3B F | GCAAAGACCGAGGGGATGAA |
| H-DNMT3B R | CCTGCCACAAGACAAACAGC |
| H-FAR2 F | AGAGACCTTTCAGGAGGCCA |
| H-FAR2 R | GAGCCGCAGATAGCAGTCAT |
| H-CCNA2 F | GAGACCCTGCATTTGGCTGT |
| H-CCNA2 R | CAGTGCCCACAAGCTGAAGT |
