# Supplementary material for: Identification of a m6A-immune-related risk model for predicting prognosis, immune microenvironment, and drug responses in acute myeloid leukemia
Source: Sci Rep. 2025 Nov 3;15:38306. doi: 10.1038/s41598-025-22002-5 (PMC12583824; doi:10.1038/s41598-025-22002-5)
Supplement: Supplementary file 1 — Supplementary Material 1 [file 41598_2025_22002_MOESM1_ESM.zip › Supplementary_Material/Table S4.docx]

**Table S4.** M6A cluster1 vs cluster2 differential biology pathway

| **Biology pathway** | **logFC** | **AveExpr** | **t** | **P.Value** | **adj.P.Val** | **B** |
| --- | --- | --- | --- | --- | --- | --- |
| ESTROGEN_RESPONSE_EARLY | -0.5399 | -0.01951 | -9.70022 | 6.26E-16 | 2.63E-14 | 25.68962 |
| APICAL_JUNCTION | -0.57541 | -0.03157 | -9.38507 | 2.99E-15 | 6.27E-14 | 24.14125 |
| MYOGENESIS | -0.59223 | -0.01419 | -8.40005 | 3.89E-13 | 5.45E-12 | 19.32307 |
| ESTROGEN_RESPONSE_LATE | -0.51969 | -0.02407 | -8.13413 | 1.43E-12 | 1.51E-11 | 18.03375 |
| INFLAMMATORY_RESPONSE | -0.57944 | -0.03147 | -8.02338 | 2.47E-12 | 2.07E-11 | 17.49893 |
| REACTIVE_OXYGEN_SPECIES_PATHWAY | -0.55769 | -0.03251 | -7.48208 | 3.41E-11 | 2.39E-10 | 14.90755 |
| SPERMATOGENESIS | 0.544041 | 0.027789 | 7.169103 | 1.53E-10 | 9.15E-10 | 13.43038 |
| EPITHELIAL_MESENCHYMAL_TRANSITION | -0.48719 | 0.005431 | -6.87354 | 6.19E-10 | 3.25E-09 | 12.05297 |
| CHOLESTEROL_HOMEOSTASIS | -0.57677 | -0.03053 | -6.66377 | 1.65E-09 | 7.71E-09 | 11.08728 |
| HEME_METABOLISM | -0.41634 | -0.03954 | -6.52274 | 3.18E-09 | 1.34E-08 | 10.44421 |
| E2F_TARGETS | 0.568058 | 0.014218 | 6.379816 | 6.15E-09 | 2.34E-08 | 9.797904 |
| INTERFERON_ALPHA_RESPONSE | -0.45842 | -0.03865 | -6.36156 | 6.68E-09 | 2.34E-08 | 9.715755 |
| COAGULATION | -0.48782 | -0.03192 | -6.29033 | 9.26E-09 | 2.99E-08 | 9.39617 |
| INTERFERON_GAMMA_RESPONSE | -0.45462 | -0.03122 | -6.1967 | 1.42E-08 | 4.25E-08 | 8.978363 |
| XENOBIOTIC_METABOLISM | -0.37826 | -0.01216 | -6.16384 | 1.65E-08 | 4.61E-08 | 8.832383 |
| UV_RESPONSE_UP | -0.42067 | -0.02 | -6.07177 | 2.49E-08 | 6.55E-08 | 8.425102 |
| TNFA_SIGNALING_VIA_NFKB | -0.50395 | -0.02445 | -5.86711 | 6.23E-08 | 1.54E-07 | 7.529709 |
| P53_PATHWAY | -0.37564 | -0.0207 | -5.76062 | 9.98E-08 | 2.33E-07 | 7.069514 |
| G2M_CHECKPOINT | 0.407234 | 0.014184 | 5.590302 | 2.10E-07 | 4.65E-07 | 6.342093 |
| IL2_STAT5_SIGNALING | -0.30842 | -0.00844 | -5.52461 | 2.80E-07 | 5.87E-07 | 6.06446 |
| ALLOGRAFT_REJECTION | -0.35648 | -0.01245 | -5.29759 | 7.39E-07 | 1.48E-06 | 5.118361 |
| MYC_TARGETS_V1 | 0.412207 | 0.006992 | 5.21166 | 1.06E-06 | 2.03E-06 | 4.765896 |
| PI3K_AKT_MTOR_SIGNALING | -0.32029 | -0.02361 | -4.69202 | 8.92E-06 | 1.63E-05 | 2.706376 |
| HYPOXIA | -0.29857 | -0.00804 | -4.58235 | 1.38E-05 | 2.41E-05 | 2.288729 |
| TGF_BETA_SIGNALING | -0.29861 | -0.01761 | -4.25936 | 4.77E-05 | 8.01E-05 | 1.096252 |
| COMPLEMENT | -0.2979 | -0.02721 | -4.13125 | 7.68E-05 | 0.000124 | 0.639661 |
| KRAS_SIGNALING_UP | -0.23748 | -0.00494 | -4.1087 | 8.35E-05 | 0.00013 | 0.560283 |
| MTORC1_SIGNALING | 0.248489 | 0.005757 | 3.660972 | 0.00041 | 0.000615 | -0.95026 |
| PROTEIN_SECRETION | 0.203319 | 0.003832 | 2.816506 | 0.005888 | 0.008527 | -3.42559 |
